# Strategies to increase uptake of voluntary medical male circumcision among men aged 25–39 years in Nyanza Region, Kenya: Results from a cluster randomized controlled trial (the TASCO study)

**DOI:** 10.1371/journal.pone.0276593

**Published:** 2023-02-03

**Authors:** Jonathan M. Grund, Frankline Onchiri, Edward Mboya, Faith Ussery, Paul Musingila, Spala Ohaga, Elijah Odoyo-June, Naomi Bock, Benard Ayieko, Kawango Agot

**Affiliations:** 1 Division of Global HIV & TB, Center for Global Health, Centers for Disease Control and Prevention, Atlanta, Georgia, United States of America; 2 Core for Biomedical Statistics, Seattle Children’s Research Institute, Seattle, Washington, United States of America; 3 Impact Research and Development Organization, Kisumu, Kenya; 4 Division of Global HIV & TB, Center for Global Health, Centers for Disease Control and Prevention, Kisumu, Kenya; International AIDS Vaccine Initiative, UNITED STATES

## Abstract

**Introduction:**

Voluntary medical male circumcision (VMMC) for HIV prevention began in Nyanza Region, Kenya in 2008. By 2014, approximately 800,000 VMMCs had been conducted, and 84.9% were among males aged 15–24 years. We evaluated the impact of interpersonal communication (IPC) and dedicated service outlets (DSO) on VMMC uptake among men aged 25–39 years in Nyanza Region.

**Materials and methods:**

We conducted a cluster randomized controlled trial in 45 administrative Locations (clusters) in Nyanza Region between May 2014 and June 2016 among uncircumcised men aged 25–34 years. In arm one, an IPC toolkit was used to address barriers to VMMC. In the second arm, men were referred to DSO that were modified to address their preferences. Arm three combined the IPC and DSO arms, and arm four was standard of care (SOC). Randomization was done at Location level (11–12 per arm). The primary outcome was the proportion of enrolled men who received VMMC within three months. Generalized estimating equations were used to evaluate the effect of interventions on the outcome.

**Results:**

At baseline, 9,238 households with men aged 25–39 years were enumerated, 9,679 men were assessed, and 2,792 (28.8%) were eligible. For enrollment, 577 enrolled in the IPC arm, 825 in DSO, 723 in combined IPC + DSO, and 667 in SOC. VMMC uptake among men in the SOC arm was 3.2%. In IPC, DSO, and combined IPC + DSO arms, uptake was 3.3%, 4.5%, and 4.4%, respectively. The adjusted odds ratio (aOR) of VMMC uptake in the study arms compared to SOC were IPC aOR = 1.03; 95% CI: 0.50–2.13, DSO aOR = 1.31; 95% CI: 0.67–2.57, and IPC + DSO combined aOR = 1.31, 95% CI: 0.65–2.67.

**Discussion:**

Using these interventions among men aged 25–39 years did not significantly impact VMMC uptake. These findings suggest that alternative demand creation strategies for VMMC services are needed to reach men aged 25–39 years.

**Trial registration:**

clinicaltrials.gov identifier: NCT02497989.

## Introduction

Male circumcision (MC) has been demonstrated to reduce female-to-male transmission of HIV by approximately 60% in randomized controlled trials [1–3]. Consequently, the World Health Organization (WHO) and Joint United Nations Programme on HIV/AIDS (UNAIDS) recommended that MC should be implemented as a new, effective HIV prevention intervention in settings with high HIV and low MC prevalence [4]. Fourteen countries in Eastern and Southern Africa were determined to be priority countries for MC implementation for HIV prevention [4]. Kenya is one of the priority countries due to its high national HIV prevalence (7.1% in 2007) and low MC coverage, especially in regions most affected by HIV [5–8]. In traditionally non-circumcising areas in the former Nyanza Province, HIV prevalence at the start of the circumcision program in 2008 was 15%, and MC coverage was considerably lower (46.4%) than the national average of 84% [9]. HIV prevalence in Kenya was significantly higher among uncircumcised men aged 15–64 years than circumcised men (16.9% vs. 3.1%) [10].

Kenya’s Ministry of Health began implementing voluntary medical male circumcision (VMMC) for HIV prevention services in 2008, with a four-year goal of increasing the proportion of circumcised men aged 15–49 years to 94%, which was estimated to be 860,000 males nationwide, and the goal for Nyanza Province was to increase circumcision coverage from 46.4% to 80% [5, 11]. Mathematical modeling estimated that performing 570,000 VMMCs in Nyanza Province up to the year 2025 would avert 70,000 new HIV infections and save approximately $371 million in averted HIV treatment costs [12]. From 2008–2013, almost 800,000 VMMCs were performed in prioritized scale-up districts in Nyanza, Nairobi, Rift Valley, and Western Provinces, and the overall proportion of males aged 15–64 years who self-reported being circumcised increased from 85.0% to 91.2% in 2012 [6, 7]. While Kenya experienced tremendous VMMC scale-up across all age groups from 2007–2012, increases in VMMC uptake gradually decreased in older age groups [6].

Studies have demonstrated that to maximize the immediacy of VMMC’s impact on HIV incidence, VMMC must be provided to HIV-negative males in the age groups where most new infections are likely to occur [12–14]. Though HIV incidence is high among men aged ≥25 years, Kenya’s VMMC program has largely appealed to younger males, data from the Kenya AIDS Indicator Survey (KAIS) 2012 suggests that a large proportion (84.9%) of HIV-negative males who were circumcised from 2010 to 2012 were aged 15–24 years [6]. Similarly, according to programmatic data from one of Kenya’s VMMC implementing partners, out of the 501,040 VMMCs performed from 2008 to 2015, 89.3% (447,266) were among males aged 10–24 years. Creating and increasing demand for VMMC among men aged ≥25 years has been a notable challenge in Kenya and other VMMC priority countries as well [15–17]. Therefore, additional strategies are needed that address barriers to VMMC uptake specifically for men aged ≥25 years to have the most immediate impact on HIV incidence.

In response to barriers of VMMC uptake identified by older men, we designed a cluster randomized trial to determine the effect of two interventions aimed at increasing VMMC uptake among men aged 25–39 years (S1 Protocol). These interventions included interpersonal communication (IPC) and dedicated service outlets (DSO), which were delivered separately and together. We hypothesized that among uncircumcised men aged 25–39 years, these interventions would significantly increase the proportion of men who take up VMMC compared to men randomized to routine service delivery and demand creation.

## Materials and methods

Target, Speed, Coverage (TASCO) of VMMC scale-up was an implementation science study conducted by Impact Research and Development Organization (IRDO) and US Centers for Disease Control and Prevention (CDC) between May 2014 and September 2016. Ethical approval was obtained from the Kenyatta National Hospital/University of Nairobi institutional review board (P36/02/2013) and US CDC’s institutional review board (protocol number 6456). The study is registered at clinicaltrials.gov (NCT02497989). Due to administrative delays in study registrations, this study was entered on clinicaltrials.gov after participant enrollment began. The authors confirm that all ongoing and related trials for this intervention are registered.

The trial was conducted in 11 sub-counties of the former Nyanza Province [referred to here as ‘Nyanza Region’] of western Kenya (S1 Fig) between April 2014 and September 2016 among uncircumcised men aged 25–39 years. Out of 164 administrative locations in the eleven districts where IRDO worked, we systematically selected 45 non-contiguous ones, which were the study clusters and served as the units of randomization. Locations are currently the fourth largest subnational administrative units in Kenya after County, Sub-county, and Ward. Each selected Location was randomized to one of the four study arms prior to study implementation: IPC, DSO, combined IPC + DSO, and the routine standard of care arm. The 45 Locations were entered into Stata v14.0 (StataCorp LP, College Station, USA) by the lead statistician, and a randomization schedule produced using a random number generator. Using simple random sampling, roughly equal number of Locations were allocated to each study arms (IPC 11, DSO 11, IPC+DSO 12 and Control 11). We then randomly selected 209 villages from the systematically selected 45 non-contiguous Locations to participate in the study and conducted a household enumeration to identify households with men in our targeted age range (25–39 years). The primary outcome was the proportion of men aged 25–39 years who took up VMMC within three months of enrollment. Male research assistants (RAs) who were fluent in English and Dholuo (the predominant local dialect) were recruited and trained on study procedures, including physical verification and documentation of circumcision status, among other topics. Men interviewed in the randomly selected homes who met the eligibility criteria (aged 25–39 years, resident of the study village with the intention of living in the same village for the next 9 months, and uncircumcised) were administered a written informed consent for study participation and physical verification of circumcision status. Eligible men who consented to physical verification of their circumcision status identified a private location of their choice for this brief examination. Consenting men were then interviewed by the RAs on demographics (age, religion, education level, employment status and marital status), and mobilized individually for VMMC according to one of the four groups their household area had been assigned (see *Intervention Groups* below).

Participants who presented to a VMMC facility within three months of their study enrollment date were identified by the names provided at enrollment, study referral coupon if available, national identification with photo, and/or cell phone number. RAs were stationed at all health facilities in the study service provision facilities to identify participants who presented for VMMC by confirming identification with a master log. After the three-month period, RAs called all enrolled men not documented to have been seen at a facility to ask if they had undergone VMMC. For those who said yes, a home visit was conducted to confirm circumcision status. Using these two methods of determining recent circumcision, the proportion of those enrolled who underwent VMMC in each intervention arm was compared to VMMC uptake among men in the routine standard of care clusters.

## Intervention groups

Those residing in the intervention clusters (IPC, DSO, and combined IPC + DSO) were reached with the respective intervention within two weeks of study enrollment. For the IPC arm, we developed a toolkit consisting of common barriers and facilitators related to VMMC based on a literature review and from TASCO’s formative data on VMMC uptake in Nyanza Region. The toolkit was used to facilitate one-on-one discussions between RAs and uncircumcised men, which permitted the RAs to respond to and address personal concerns about circumcision communicated by uncircumcised men. The topics covered in the IPC toolkit included accurate information about pain during and after VMMC surgery, likelihood of complications, dealing with recommended sexual abstinence post-surgery, etc. In the DSO arm, the physical layout of included VMMC sites was modified to accommodate older men’s preferences. The DSO arm was based on literature from sub-Saharan African countries implementing VMMC that suggested that older men seeking VMMC services preferred separate waiting areas at clinics, male-only service providers, shorter waiting times, and flexible service delivery hours. Thus, the study services provided in DSO locations included male-only providers, separate waiting areas, private access to the theater in some facilities, and flexible hours for selected facilities, (e.g., two weekends a month or two evenings a week to serve only men ≥ 25 years of age). RAs went to the homes of eligible men in the Locations randomized to the DSO arm and referred them to these DSO sites. In the combined IPC + DSO arm, we used the toolkit to address men’s reasons for not going for circumcision and referred them to DSO sites. In the routine standard of care arm, routine demand creation activities under the VMMC program continued [16, 17]. All participants in the four arms were advised to undergo VMMC within three months of their enrollment and were informed that they would also receive a text message reminder two months after enrollment into the study in case they had not already sought VMMC.

## Statistical methods

### Sample size calculation

A previous demand creation and HIV community-based study found that circumcision prevalence among men aged 25–49 years in sub-Locations included in our study area was 35.6% [18], and program data estimated prevalence to be 30% for men aged 25–39 years. The anticipated rate of men declining to take part in the study was 15%, and the expected loss to follow-up was estimated at 15%. Using these estimates together with an intra-cluster correlation coefficient (ICC) of 0.005, and a design effect of 2.5 in sample size formula for cluster studies [19], we estimated that 4,932 men would be needed in order to be able to detect a difference of at least a 33.3% in VMMC uptake among eligible men between each of the intervention arms and the standard of care arm with 80% statistical power. Due to recruitment and logistical challenges, only 2,792 participants were enrolled for the study.

### Statistical analyses

We summarized baseline characteristics at individual level stratified by study arm. Categorical variables were summarized using counts and proportions with Rao-Scott Chi-square [20] comparison methods to assess potential differences between study arms. We evaluated the effect of the intervention on the odds of VMMC uptake by comparing the proportion of VMMC uptake in each of the intervention arms to that in the routine standard of care arm using generalized estimating equations (GEE) logistic regression to account for the clustering on men within the study Locations with robust standard errors and the assumption of equal correlation between men in the same cluster [21, 22]. In a multivariable GEE logistic model, we assessed the effect of the intervention on VMMC uptake while adjusting for the chance imbalances of the baseline covariates between study arms. Variables with global p-value<0.25 in the unadjusted analysis were included in the adjusted analysis [23] plus age which is a known confounder. Unadjusted and adjusted odds ratios (and their corresponding 95% confidence intervals (CIs)) of the association between interventions and the proportions of men that come for VMMC are presented. The GEE logistic regression models were fitted using Stata v14.0. All statistical tests were done at 5% level of significance.

## Results

**Fig 1:**
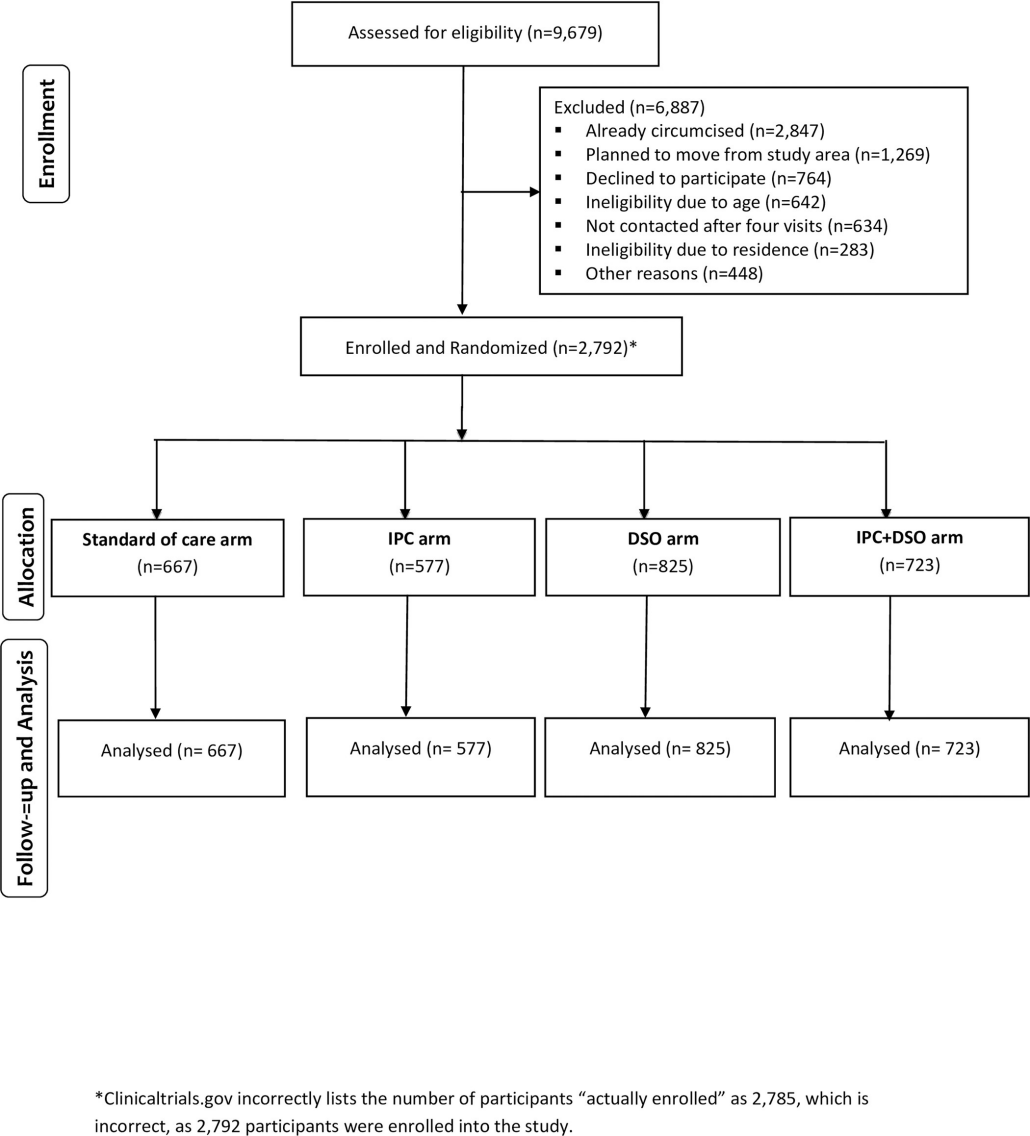
Participant screening, enrollment, and follow-up. *Clinicaltrials.gov incorrectly lists the number of participants “actually enrolled” as 2,785, which is incorrect, as 2,792 participants were enrolled into the study.

Between May 2014 and June 2016, 9,238 households with men aged 25–39 years were enumerated, from which 9,679 men were screened and 2,792 (28.8%) enrolled in the study. The main reasons for men not enrolling included already being circumcised (41.3%), planning to move away from the study area (18.4%), ineligibility due to age (9.3%), not found after four household visits (9.2%), and ineligibility due to residence (4.1%). In addition, 764 participants (11.1%) were eligible but refused to participate. Among the 2,792 uncircumcised men who were eligible and enrolled, 577 were in the IPC arm, 825 in DSO arm, and 723 in the combined IPC + DSO arm and 667 in the standard of care Locations (Fig 1).

In all arms at baseline, most uncircumcised men were married, Christian, had completed up to primary level education, and employed. Baseline socio-demographic characteristics of uncircumcised men are presented by study arm in Table 1. Baseline circumcision prevalence has been reported elsewhere [23].

**Table 1:** Baseline socio-demographic characteristics of uncircumcised men by study arm.

	Study arm			
Factor	Standard of Care (N = 667) n (%)	DSO (N = 825) n (%)	DSO+IPC (N = 723) n (%)	IPC (N = 577) n (%)
**Age (in years)**				
25–29	261 (39.1%)	307 (37.2%)	283 (39.1%)	228 (39.5%)
30–34	215 (32.2%)	269 (32.6%)	246 (34.0%)	200 (34.7%)
35–39	191 (28.6%)	249 (30.2%)	194 (26.8%)	149 (25.8%)
**Marital status**				
Single	52 (7.8%)	82 (9.9%)	72 (10.0%)	72 (12.5%)
Married	598 (89.7%)	733 (88.8%)	616 (85.2%)	484 (83.9%)
Divorced/Sep/Widowed	17 (2.5%)	10 (1.2%)	35 (4.8%)	21 (3.6%)
**Religion**				
Christian	661 (99.1%)	820 (99.4%)	718 (99.3%)	572 (99.1%)
Non-Christian	6 (0.9%)	5 (0.6%)	5 (0.7%)	5 (0.9%)
**Education**				
Primary or below	478 (71.7%)	464 (56.2%)	531 (73.4%)	399 (69.2%)
Secondary	141 (21.1%)	256 (31.0%)	145 (20.1%)	129 (22.4%)
College/University	48 (7.2%)	105 (12.7%)	47 (6.5%)	49 (8.5%)
**Employment**				
Employed	377 (56.5%)	562 (68.1%)	379 (52.4%)	319 (55.3%)
Unemployed	290 (43.5%)	263 (31.9%)	344 (47.6%)	258 (44.7%)

The proportion of VMMC uptake documented at the service delivery sites or via home visits among enrolled men aged 25–39 years within 3 months of enrollment in the standard of care arm was 3.2%. In the IPC, DSO, and the combined IPC + DSO arms, VMMC uptake was 3.3%, 4.5%, and 4.4%, respectively. There were no significant differences in odds of VMMC uptake across the intervention arms when compared to the standard of care arm. The adjusted odds ratio (aOR) and their corresponding 95% confidence intervals (95% CI) were: IPC aOR = 1.03, 95% CI: 0.50–2.13); DSO aOR = 1.31, 95% CI: 0.67–2.57; and IPC + DSO combined aOR = 1.31, 95% CI: 0.65–2.67 (Table 2). Across arms, there was no observed difference in odds of VMMC uptake among men aged 25–29 (aOR = 1.47; 95% CI: 0.92–2.35) or men aged 30–34 (aOR = 1.36; 95% CI: 0.80–2.32) compared to men aged 35–39, adjusting for marital status, religion, education, and employment.

**Table 2:** Analysis of the effects of the interventions on uptake of VMMC three months after enrollment.

		Unadjusted			Adjusted*		
Study Arm	Number of men circumcised** (%)	OR	95% CI	p-value	OR	95% CI	p-value
Standard of care	21/667 (3.2)	1 [Ref]			1 [Ref]		
IPC	19/577 (3.3)	1.05	(0.54–2.04)	0.882	1.03	(0.50–2.13)	0.931
DSO	37/825 (4.5)	1.33	(0.73–2.43)	0.347	1.31	(0.67–2.57)	0.434
IPC + DSO	32/723 (4.4)	1.29	(0.70–2.39)	0.409	1.31	(0.65–2.67)	0.451

*Adjusted for baseline factors: age, marital status, religion, education and employment.

** Men who underwent VMMC who were verified to have enrolled in the study via referral coupon, names, national identification, and/or phone number

## Discussion

This cluster randomized trial did not provide sufficient evidence that targeted demand creation strategies for men aged 25–39 years were effective at significantly increasing the odds of VMMC uptake among men aged 25–39 years in the Nyanza Region of western Kenya. Overall VMMC uptake across each of the four study arms ranged from 3.2%– 4.5%. It is likely that the DSO and IPC + DSO arms had some effect, but the smaller than expected sample size prevented the detection of these effects. Based on the original sample size calculations, we estimated that 1,480 men in intervention arms and 370 in the standard of care arm would be needed to provide 80% power to detect any intervention effect. Only 109 men of the expected 1,850 men underwent VMMC. Due to higher VMMC prevalence than anticipated in study Locations, only 2,792 participants were enrolled for the study, as many men were circumcised and therefore ineligible (n = 2,847). Logistical challenges and funding limitations prevented the study team from expanding the study scope to increase the number of enrolled participants and study power.

While mathematical modeling has suggested that circumcising men aged 20–34 years in several VMMC priority countries in sub-Saharan Africa would result in a more immediate reduction in HIV incidence [12, 15], alleviating key individual- and facility-based barriers in our study did not increase VMMC uptake among men aged 25–39 years in Nyanza Region, Kenya.

VMMC has been successful in Kenya in the non-traditionally circumcising communities in the former Nyanza Province with over 1.1 million males circumcised by 2016, which represents 132% of the initial target set in 2011 [10]. However, 47% of clients circumcised for HIV prevention in Kenya through the United States President’s Emergency Plan for AIDS Relief (PEPFAR) support in fiscal year 2015 were aged 10–14 years, 48% were aged 15–29 years, and only 5% were aged ≥30 years [internal PEPFAR data], so the challenges experienced in this study are consistent with those facing the broader VMMC program in Kenya. VMMC demand creation focuses on uncircumcised male clients aged 15–29 years, so it is possible that the messages promoted in the TASCO study were understood to be targeting the same priority age groups.

Kenya is also a leader in the scale-up of VMMC for HIV prevention, as Kenya’s Ministry of Health first launched VMMC services in 2008 following development of the National Guidance on Male Circumcision and Kenya National Strategy for Voluntary Medical Male Circumcision [24–26]. It is possible that men aged 25–39 years have known about VMMC for years and those who remain uncircumcised have decided not to pursue it and are therefore less likely to be influenced by additional demand creation interventions. One analysis of the KAIS 2012 data found that 72.1% of uncircumcised HIV-negative men knew that VMMC was partially protective and 96.0% knew that circumcised men still needed to use a condom to prevent HIV infection [6].

The low uptake of VMMC across all study arms suggests that men aged 25–39 years were likely not adequately encouraged to pursue VMMC. One recent study about post-operative follow-up in former Nyanza Province found that 98.8% of VMMC clients who returned for post-operative follow-up lived within 5km of a VMMC site, and men living farther than 5km were significantly less likely to attend VMMC post-operative follow-up visits [27]. Each Location had only one study-designated facility, which meant that it is possible that some participants were referred to facilities that were more than 5km away from their homes. This may have resulted in increased distances for some men to travel to access a study-designated facility, which may have limited VMMC uptake. Another recent study in Tanzania found that men aged 20–34 years living in a region where VMMC scale-up was more mature were less likely to accept VMMC after being exposed to an innovative demand creation campaign [28]. Given that Kenya was the first country to launch a VMMC program in 2008 and over 500,000 VMMCs had already been performed by the time this study began in 2014, it is also plausible that these interventions would have been more successful in settings with nascent VMMC programs.

Another VMMC demand creation trial took place in Nyanza Region for uncircumcised men aged 25–49 years offered food vouchers of varying amounts conditional on getting circumcised [18]. VMMC uptake increased significantly in the two groups with the highest incentives (to 6.6% in the $8.75 arm, and to 9.0% in the $15.00 arm, compared with 1.6% in the control group with no food vouchers), though the absolute increases were modest. This suggests that economic barriers play a role in at least some men’s decisions to seek circumcision. Additional studies are needed to determine the best balance of demand creation interventions that integrate components of interpersonal communication, modified facility layout, and financial incentives.

Our trial had several limitations. Firstly, two of the intervention arms showed some increased VMMC uptake, but the low sample size did not allow us to detect these effects, thus potentially large effects cannot be ruled out. Secondly, the short time of the observation (three months) may have limited the period for men, the majority of whom were employed hence needed permission to be away, to become circumcised in each of the study arms. Thirdly, some men who received circumcision may have been in the study, but RAs were unable to verify their enrollment or to find them at home to document circumcision status. And finally, participants in all four study arms received a text message reminder about VMMC, so the differential impact of this component is unknown.

In conclusion, our study showed that due to low service uptake across all study arms, enhanced interpersonal communication and modified facilities did not significantly increase VMMC uptake among men aged 25–39 years in Nyanza Region, Kenya. Additional demand creation strategies need to be designed and evaluated to determine how best to encourage older men to seek VMMC for HIV prevention, especially in settings with mature VMMC programs. HIV prevention programs should explore whether men aged 20–39 years in Nyanza Region are not interested in VMMC regardless of the intervention types, and if males aged 10–19 years should be the focus of the limited HIV prevention resources.

## Supporting information

S1 Checklist CONSORT 2010 checklist of information to include when reporting a cluster randomised trial. (DOCX)

S1 Fig TASCO locations by randomization into the 4 study arms. This map was generated by the implementing partner, IRDO, and was previously published in another manuscript related to this study: https://journals.plos.org/plosone/article/figure?id=10.1371/journal.pone.0185872.g001.
(DOC)

S1 Protocol (DOCX)
