# Supplementary material for: Strategies to increase uptake of voluntary medical male circumcision among men aged 25–39 years in Nyanza Region, Kenya: Results from a cluster randomized controlled trial (the TASCO study)
Source: PLoS One. 2023 Feb 3;18(2):e0276593. doi: 10.1371/journal.pone.0276593 (PMC9897540; doi:10.1371/journal.pone.0276593)

Figure 1: TASCO Locations by randomization into the 4 study arms

BUSIA

128

143

131

140

126

121

119

123

132

124

138

BUTERE/MUMIAS

NANDI

141

134

142

117

120 **SIAYA**

129

139

116

127

125

130

115

144

122

VIHIGA

8

4 6 2

4 11

136

5

133

3

118

135

137

54

55

58 56

44

40 45 51 39

42

9 **BONDO**

**KISUMU WEST** 5049

92 57 59

53 52

41 43 46

7 97

1

90

10

94 60

60

# KISUMU EAST

38

37 48

# RARIEDA

93

91 95

**Lake Victoria**

47 NYANDO

37

96

KERICHO

162

160

150

164

RACHUONYO

# SUBA

159

146

161

159

155

151

145

157 18

15

22 32

25

20 15

147

156

154

152

149

# SUBA

153

163

34

158

19

29

13

# HOMA BAY

33 21

17

26

31 12 103

32

25

24

23

107

CENTRAL KISII

NYAMIRA

148

# NDHIWA

16

101

36

89 68

62

27

14

28

35 30

109

| **Legend** | |
| --- | --- |
|  | CONTROL |
|  | |
|  | DSO |
|  | |
|  | IPC |
|  | |
|  | IPC & DSO  IPC & DSO |
|  |  |
| 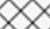 | Ineligible locations |

75 100

105

102

110

GUCHA

73

72

61 **NYATIKE**

108

114

# RONGO

98

106

113

99

88

67 69

63

76

74

77

79

70 80

65

83

111

71

112

104

TRANSMARA

TRANS MARA

. 64

# MIGORI

78 66 81

84 82

86

85

87

KURIA

## 0 4.5

9 18 27 36

km

1 cm = 7 km


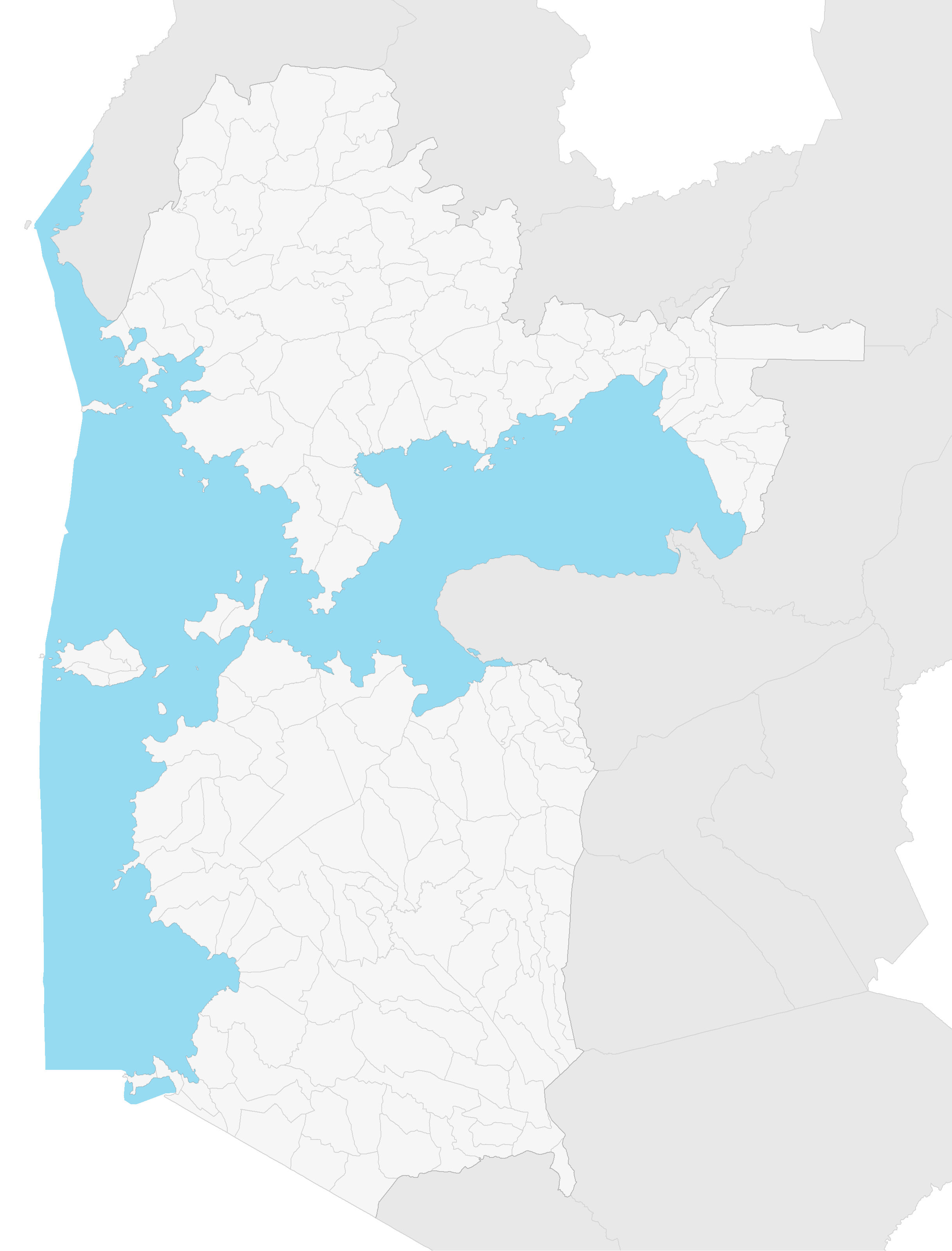

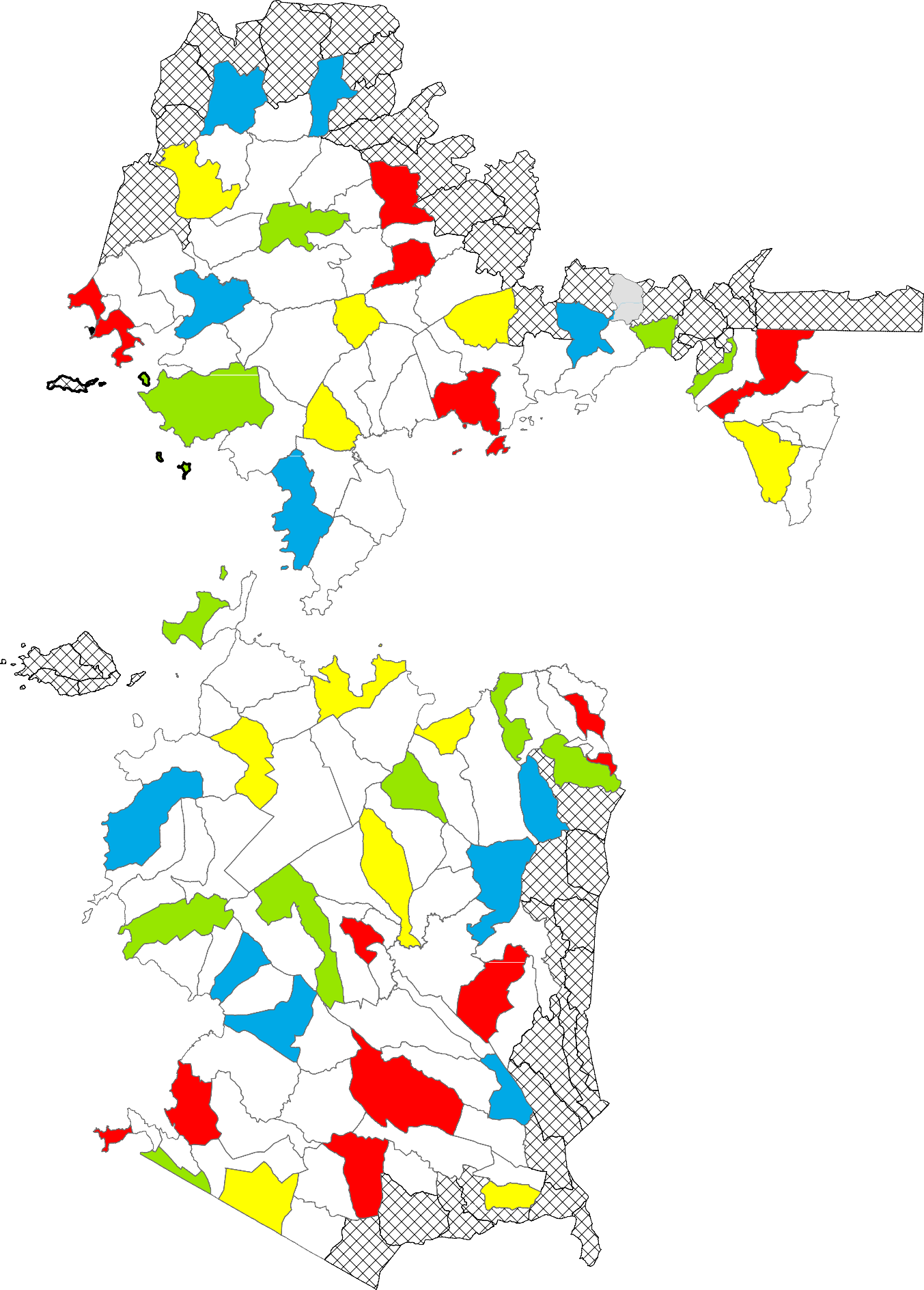

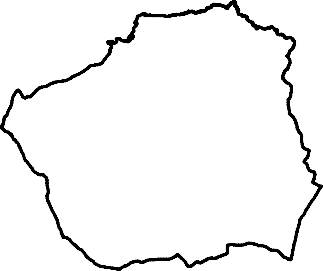

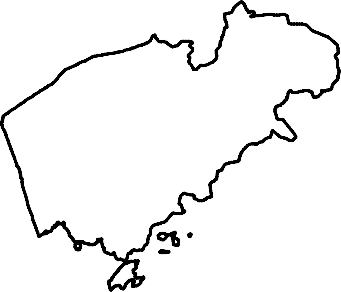

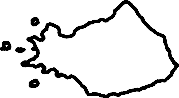

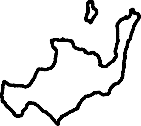

Supplement: S1 Fig — This map was generated by the implementing partner, IRDO, and was previously published in another manuscript related to this study: https://journals.plos.org/plosone/article/figure?id=10.1371/journal.pone.0185872.g001. (DOC) [file pone.0276593.s002.doc]
