# Supplementary material for: Strategies to increase uptake of voluntary medical male circumcision among men aged 25–39 years in Nyanza Region, Kenya: Results from a cluster randomized controlled trial (the TASCO study)
Source: PLoS One. 2023 Feb 3;18(2):e0276593. doi: 10.1371/journal.pone.0276593 (PMC9897540; doi:10.1371/journal.pone.0276593)
Supplement: S1 Protocol — (DOCX) [file pone.0276593.s003.docx]

#

**REF: KENYA: IMPACT/"OPERATIONS RESEARCH" GH11-005, GH000518**

**Title of Study: Male Circumcision for HIV Prevention in Kenya: Seeking Effective Strategies to Recruit Older Men.**

**Principal Investigator: Kawango Agot, PhD, MPH (Principal Investigator, IRDO)**

**Co-Principal Investigator: Jonathan Grund, MA, MPH (CDC-Atlanta)**

**Co-Investigators:**

**Eunice Omanga, MES, DrPH (Research Officer, IRDO)**

**Naomi Bock, MD, MPH (CDC-Atlanta)**

**Jacob Onyango, BA, MA, Study Coordinator, IRDO**

**Frankline Onchiri, MSPH, PhD(c) (Biostatistics, KEMRI/University of Washington)**

**June Odoyo, MBChB, MSc, PhD(c) is the Technical Adviser, HIV Prevention at CDC.**

**Ohaga Spala, MSc., PhD (Programs Manager, IRDO)**

**Samuel Mwalili, PhD (CDC, Kenya)**

**Edward Mboya, BSc, MSc (Statistics), (Senior Data Analyst) IRDO**

**Julie Ambia, BSN, MPH, PhD(c), University of Nairobi**

**Emily Zeilinski-Gutierrez, MPH, DrPH,** **CDC-Kenya**

**Donath Emusu, MBChB, MPH, DrPH, Senior Technical Advisor, CDC-Kenya**

**Clement Zeh, PhD (Director, CDC/KEMRI HIV Research Laboratory)**

**Walter Otieno Agingu, HND-Medical Laboratory Sciences (IRDO)**

**Bernard Ayieko, HND, MCHD (Coordinator, IRDO VMMC Rollout Program)**

**Boaz Otieno-Nyunya, MBChB, MMed (CDC-Kenya)**

**Dr. Duncan Odera, MBChB (VMMC Clinical Manager, IRDO)**

**Leonard Soo, BSN, MPH, DrPH (c), IRDO’s Technical Advisor, CDC-Kenya**

**Athanasius Ochieng’, MBChB, Programs Manager, Ministry of Health-Kenya**

**Ojwang’ Lusi, MBChB, MMED (Surgery) (Co-Chair, Inter-County VMMC Taskforce)**

**Consultants: Urbanus Kioko, MA, MPhil, PhD (Health Economist, UoN)**

**Harsha Thirumurthy, PhD. (Health Economist)**

**Victor Ssempijja, MSc (Biostatistician)**

**Implementing Institution: Impact Research & Development Organization (IRDO)**

**Proposed Duration: Up to 24 months (approximately 6-9 months of data collection)**

# ABSTRACT

**Background:** In Kenya, 1.4-1.6 million people are living with HIV, with an adult prevalence of 6.3%. Nyanza, one of the eight provinces, has the highest prevalence, at 13.9%. Within Nyanza, HIV prevalence in the districts occupied by the Luo - the dominant ethnic community in the province and third nationally – was 20.2% in 2008/09 (17.1% in men and 22.8% in women). Several drivers of the rapid spread of HIV in the province have been identified as, among others, lack of male circumcision. A large number of observational studies and three randomized controlled trials concurred in their findings, that voluntary medical male circumcision (VMMC) reduces men’s risk of acquiring HIV by about 60%. Following the publications of these results and subsequent endorsement by the World Health Organization and UNAIDS, Kenya rolled out VMMC services from October 2008. By June 2013, our organization had performed 241,145 circumcision procedures. However, uptake of services by older men (aged ≥25 years) who engage in behaviors that put them at higher risk of acquiring HIV has been low, at about 30%. We propose to evaluate the impact of two interventions (Inter-Personal Communication [IPC] and Dedicated Service Outlets [DSOs]) in recruiting men aged 25-39 years for VMMC services.

**Aims**: i) assess the rate of uptake of VMMC services among four categories of men: those exposed IPC, those exposed to DSO, those exposed to both IPC and DSO combined, and those receiving no intervention; ii) determine, through unlinked testing of blood from bleeding vessels from surgical wound, the proportion of men with unknown HIV status; and iii) determine the cost of providing VMMC among each of the three interventions vs. no intervention.

**Methods:** We will employ cluster-randomized controlled design. Through elimination by factors such as proximity to circumcising communities, multi-ethnic urban populations, and geographical proximity to each other, 45 administration Locations in Nyanza Province have been selected and randomized into IPC implemented alone, DSO implemented alone, IPC and DSO implemented together and control. Under IPC, we will develop a Toolkit for Addressing Barriers and Facilitators to VMMC with messages that address barriers to and reinforce facilitators of VMMC, and engage behavioral counselors to deliver intervention massages that are custom-made to address participants’ specific issues; Under DSO, selected facilities, days and hours will be set aside to serve only men aged ≥25 years; these sites will be served by older, male providers only; Under Combined IPC and DSO, the effect of both interventions implemented together will be examined; and in the Control locations, no intervention will be implemented. Altogether, 4,932 men aged 25-39 years are expected to take up VMMC during the study period, which will take up to nine months.

For aim 2, we will collect blood samples from bleeding vessels of an estimated 700 men whose HIV status is unknown, and perform unlinked test for HIV at the KEMRI/CDC HIV Laboratory in Kisumu. For aim 3, we propose to extract from our procurement, stores and accounts records expenditures for demand creation and service delivery.

To augment this study, we will nest a sub-study (sub-study 4) in all the 45 study Locations and enroll approximately 720 men who are in stable sexual relationships, together with their partners, for post circumcision follow up visits to monitor the impact of involving female partners on time to resumption of sex post-surgery.

**Table of Contents**

[ABSTRACT 3](#_Toc399487742)

[LIST OF FIGURES 6](#_Toc399487743)

[LIST OF TABLES 6](#_Toc399487744)

[LIST OF ACRONYMS / ABBREVIATIONS 7](#_Toc399487745)

[BACKGROUND AND SIGNIFICANCE 9](#_Toc399487746)

[STUDY AIMS AND JUSTIFICATION 10](#_Toc399487747)

[RESEARCH STRATEGY/APPROACH 12](#_Toc399487748)

[STUDY DESIGN 12](#_Toc399487749)

[PHASE 1: HOUSEHOLD ENUMERATION 20](#_Toc399487750)

[PHASE 2: CONDUCTING RAPID FORMATIVE STUDY (SUB-STUDY 1) 21](#_Toc399487751)

[SUB-STUDY DESIGN 21](#_Toc399487752)

[Selection of the FGD/IDI Locations and Participants 23](#_Toc399487753)

[Consenting 23](#_Toc399487754)

[Procedure of the FGD 24](#_Toc399487755)

[PHASE 3: ESTABLISHING BASELINE VMMC PREVALENCE AND PROPORTION OF ELIGIBLE MEN 24](#_Toc399487756)

[PHASE 4: IMPLEMENTING THE INTERVENTIONS 26](#_Toc399487757)

[Delivery Process of IPC+DSO Intervention 29](#_Toc399487758)

[Demand creation for all other ages 29](#_Toc399487759)

[Participant Inclusion and Exclusion Criteria 29](#_Toc399487760)

[Activities that cut across study arms: 31](#_Toc399487761)

[HIV TESTING OF PARTICIPANTS WITH UNKNOWN HIV STATUS (SUB-STUDY 1) 33](#_Toc399487762)

[Sub-study 2 Inclusion/Exclusion Criteria 34](#_Toc399487763)

[Enrollment of Sub-study Participants: 35](#_Toc399487764)

[Methods/Procedure: 35](#_Toc399487765)

[Laboratory Procedure: 37](#_Toc399487766)

[Sources of costing data 39](#_Toc399487767)

[Costing Data Analysis: Incremental cost-effectiveness ratio (ICER) estimation: 40](#_Toc399487768)

[Sensitivity Analysis 41](#_Toc399487769)

[PHASE 5: ESTABLISHING ENDLINE MC UPTAKE/ESTIMATING IMPACT OF INTERVENTIONS 41](#_Toc399487770)

[DATA MANAGEMENT PLAN 42](#_Toc399487771)

[Quality assurance of data entry 42](#_Toc399487772)

[Data storage and retention 43](#_Toc399487773)

[Data Ownership and Sharing Policy 43](#_Toc399487774)

[Data Analysis: 43](#_Toc399487775)

[Other Analyses: 44](#_Toc399487776)

[Data security 45](#_Toc399487777)

# LIST OF FIGURES

Figure 1: Sample Size Vs Percentage Increase in MC Uptake 12

[Figure 2: Maps 1-4 Showing Selection Steps](#_Toc347923534) 15

# LIST OF TABLES

[Table 1: Summary of selected Study Locations. 14](#_Toc347923532)

[Table 2: The number of Locations randomized to each study arm by district 15](#_Toc347923533)

[Table 3: Randomization schedule for selected Locations.](#_Toc347923535) ..........................................16

[Table 4: Average # of Men Aged 25-39/100 Households 17](#_Toc347923550)

[Table 5: Estimated Target Population and # of Selected Villages 18](#_Toc347923550)

[Table 6: Summary of data sources for use in costing](#_Toc347923550) 38

[Table 7: Summary Flow of Forms/Appendices 4](#_Toc347923572)6

[Table 8: Proposed Timelines for Set Up and Data Collection](#_Toc347923575) 49

# LIST OF ACRONYMS / ABBREVIATIONS

| Ab | Antibody |
| --- | --- |
| Ag | Antigen |
| CDC | Centers for Disease Control and Prevention |
| CEAC | Cost-Effectiveness Acceptability Curve |
| CHW | Community Health Worker |
| CI | Confidence Interval |
| CITI | Collaborative Institutional Training Initiative |
| CO | Clinical Officer |
| DALY | Disability Adjusted Life Years |
| DBS | Dried Blood Spot |
| DMPPT | Decision-Makers’ Program Planning Tool |
| DoD | Department of Defense |
| DSO | Dedicated Service Outlet |
| ED | Emergency Department |
| EDTA | Ethylenediaminetetraacetic Acid |
| ERC | Ethics Review Committee |
| FGD | Focus Group Discussion |
| GCP | Good Clinical Practices |
| GEE | Generalized Estimating Equations |
| HDSS | Health and Demographic Surveillance System |
| HIV | Human Immunodeficiency Virus |
| HIV-R Lab | HIV-Reference Laboratory |
| HR | Hazard ratio |
| ICC | Intra-cluster Correlation Coefficient |
| ICERs | Incremental Cost-Effectiveness Ratios |
| IDI | In-Depth Interview |
| IPC | Inter-Personal Communication |
| IPO | Infection Prevention Officer |
| IRB | Institutional Review Board |
| IRDO | Impact Research and Development Organization |
| KEMRI | Kenya Medical Research Institute |
| MC | Male Circumcision |
| MOH | Ministry of Health |
| NO | Nursing Officer |
| OR | Odds ratio |
| PEPFAR | United States President’s Emergency Plan for AIDS Relief |
| PI | Principal Investigator |
| PIN | Participant Identification Number |
| RA | Research Assistant |
| RR | Risk ratio |
| SOP | Standard Operating Procedure |
| SPSS | Statistical Package for Social Science |
| STATA | Data Analysis and Statistical Software developed by STATA Corp. Tx |
| STI | Sexually Transmitted Infection |
| UNAIDS | The Joint United Nations Programme on HIV/AIDS |
| USAID | United States Agency for International Development |
| VMMC | Voluntary Medical Male Circumcision |
| WHO | World Health Organization |

# BACKGROUND AND SIGNIFICANCE

Results of three randomized controlled trials in South Africa, Kenya and Uganda have confirmed beyond reasonable doubt that voluntary medical male circumcision (VMMC) is safe and reduces the risk of HIV acquisition in heterosexual men by approximately 60%.^[[1]](#endnote-1),^^[[2]](#endnote-2),^^[[3]](#endnote-3)^ These results supported findings from previous observational studies which also indicated that VMMC significantly reduces the odds of acquiring HIV by men.^[[4]](#endnote-4),^^[[5]](#endnote-5)^ A summary of prospective studies that enrolled HIV negative participants, followed them up and monitored the rate of new HIV infections, reported that male circumcision reduced the risk of acquiring HIV by 58% in the general population and by 71% among high risk groups, such as patients in sexually transmitted infection (STI) clinics and partners of female sex workers.^4^

The Government of Kenya began providing VMMC services in October 2008, with support from donor-funded implementing partners. IRDO is one of the VMMC implementing partners and a registered Kenyan non-governmental organization whose mission is to design, implement and evaluate public health research and programs in Kenya. Of the approximately 300,000^[[6]](#endnote-6)^ circumcisions done in Nyanza Province to date, only about 12% (our sites are at 16%) of clients are aged above 25 years, yet this is the age bracket that if circumcised would translate into an immediate impact on HIV incidence.^[[7]](#endnote-7)^ We propose to implement and evaluate the impact of two interventions – Interpersonal Communications (IPC) and Dedicated Service Outlets (DSO) – independently and jointly, on demand for VMMC by men age 25-39 years in Nyanza Province.

**Why target older men?** A meta-analysis^[[8]](#endnote-8)^ and modeling studies^7^ on male circumcision (MC) suggest that circumcising older, more sexually active men within a short period of time would have a bigger and faster population-level impact in reducing incident HIV. Hankins et al^7^ propose that since HIV incidence is highest among 25-34 year old men, circumcising men in this age bracket has the biggest benefit, whereas circumcising those aged 15-24 and 35-49 years has modest impact. Circumcising men 50+ years has little effect on HIV incidence. Thus, targeting the right age and speeding up coverage are key to making an impact on HIV incidence.

Although Kenya has set the pace of VMMC in Africa by circumcising about 500,000 men in the past 4 years,^[[9]](#endnote-9)^ the country has performed rather poorly in getting older men ≥25 to take up VMMC services. The difficulty in getting older men take up VMMC services is not unique to programs; in two recently concluded operations research in Nyanza, ^[[10]](#endnote-10),^ ^[[11]](#endnote-11)^ the median age of the 4,363 study participants was 20 and 21 years. While Kenya anticipates circumcising 860,000 men aged 15-49 years by 2013/14,^[[12]](#endnote-12),^^[[13]](#endnote-13)^  it is clear that getting older men remains a challenge. New strategies to make VMMC appeal to older men must therefore be conceived, implemented and evaluated. We intend to evaluate the impact of inter-personal communication and dedicated VMMC service outlets on uptake from an average of 30% to 40%.

While the increase from 30% to 40% may seem modest, the high risk of HIV associated with being uncircumcised in this area means that doubling MC could have a significant public health impact in terms of the number of HIV infections averted. Most males getting VMMC in Kenya have a median age of 17 years. In contrast, the uptake of VMMC by the older males remains low, being about 30% (unpublished data from a demographic surveillance survey in one of the districts in Nyanza Province) while they are more likely than younger men to acquire HIV. Modeling studies by Nagelkerke, 2008^[[14]](#endnote-14)^; Hankins 2009^7^; Njeuhmeli, 2011^[[15]](#endnote-15)^ have demonstrated a strong impact of increasing VMMC coverage in the long-term on the number of HIV infections averted, especially in regions with generalized HIV epidemics. They have shown that increasing uptake of male circumcision among adults (15 – 49 years) is associated with a simultaneous increase in the cumulative number of new infections averted. For example, in Nyanza province, it has been estimated that by performing 380,000 VMMCs between 2011 – 2015, about 100,000 HIV infections would be averted, while 570,000 VMMCs performed between 2011 and 2015 would avert 700,000 HIV infections. This implies that even a modest change in the additional number of VMMCs attained could result into a considerable increase in the number of HIV infections averted.

**Why interpersonal communication (IPC)?** IPC is a strategy that involves communication between/among a few people who are in close proximity and have a shared interest. It is a one-on-one or small group communication to discuss, educate, sensitize, influence, persuade, reason with peers, friends, family, workmates, and so on. This is done with a purpose of winning them for or against an idea or action. A large body of literature,^10,^^[[16]](#endnote-16),^^[[17]](#endnote-17),^^[[18]](#endnote-18),^^[[19]](#endnote-19),^^[[20]](#endnote-20),^^[[21]](#endnote-21),^^[[22]](#endnote-22)^ indicates that key barriers to VMMC among men in Nyanza include pain, fear of complications, concern over how to feed self and family during the healing period, and perception that the penis shrinks or sexual libido and/or performance diminishes after surgery. Other barriers include VMMC being viewed as against culture or religion, the recommended period of sexual abstinence perceived as being too long, unease with lining up for services with young people, and concerns over being circumcised by young and/or female providers. However, circumcision being a component of some religions could also be a reason for some men seeking circumcision. These studies also reported that most of those who take up VMMC have been influenced by peers, those who have gone through circumcision, female sexual partners, parents, older brothers, community elders, political and religious leaders, and school/college instructors. Three of the studies^16-18^ point to strong perception that VMMC is intended for younger men <21 years. The perception is that older men are in stable relationships hence insulated from HIV. This indicates that people still have personal questions and concerns that they want addressed before making a decision for VMMC. This calls for settings tailored to favor open discussion on barriers to service uptake. We intend to engage “influencers” of decisions around VMMC, such as satisfied clients, female partners, community leaders, community health workers, and older siblings. We will build their capacity to recruit older men through open, clear, concise and repetitive dialogue in the comfort of their homes.

**Why Dedicated Service Outlets (DSOs)?** There is growing anecdotal reports from implementing partners in Kenya that one of the key barriers older men cite for not taking up VMMC services is that they are uncomfortable being made to queue with young boys for fear of being labeled promiscuous given circumcision has largely been marketed as a strategy for preventing HIV and STIs. Similar reservations have also been expressed in a study in Tanzania,^[[23]](#endnote-23)^ where male respondents pointed out that VMMC services often do not take into account social status of potential care-seekers, specifically age and gender status. For example, there were concerns that older men are circumcised by young women, and sometimes wait in line with children and youth. Additionally, young men reported feeling ashamed to be served by women, to expose their nudity, be touched, and so on; they also fear sexual arousal if service providers are young female.

In response to the concern over age-mixing and female providers, we intend to set up special clinics to serve the older population (25+ years) that are friendly and with flexible working days and hours. In these clinics, we will engage mature, male service providers only, who are within/close to the same age bracket as the participants. If shared with other services, the designated clinics will strive to ensure discretion and privacy to the clientele. Where designated clinics are not feasible, we will set aside days for older men, in addition to providing services to older men over the weekends, in the evenings and during public holidays.

# STUDY AIMS AND JUSTIFICATION

***Aim 1: Assess the rate of uptake of VMMC services by men aged 25-39 years exposed to Inter-Personal Communication (IPC) vs. Designated (older men only) Service Outlets (DSO) vs. IPC and DSO interventions combined vs. no intervention.***

Justification: Since Kenya launched VMMC in November 2008, IRDO has circumcised 241,145 men as at June 2013, aggregated by age as follows: 88% under 24 years, 12% 25-39 years, and 2% ≥40 years. IRDO’s performance is relatively better than the provincial rate, which stands at under 10%, a clear demonstration that creative strategies to create demand for VMMC among older men are urgently needed if the country is to experience the immediate benefit of VMMC on HIV incidence. We will register all participants coming for VMMC services at our sites and document their assigned arm and date of circumcision. At enrollment, we will capture the details of each participant enrolled in the study and enter them into a Participants Master Log (Appendix 14), which comprise of all enrolled participants at baseline. The details will include: participant’s 3 names, national ID number, age, location, village, telephone number (of the participant or close friend) and date of enrollment. At endline, we will extract program data on MC uptake among clients aged 25-39 years in non-study VMMC health facilities and triangulate with those registered in the Participants Master Log as having been circumcised. The purpose of this comparision is to capture study participants who may have been circumcised in non-study health facilities (further discussion is on page 41).

***Aim 2: Determine through testing of unlinked de-identified blood from bleeding vessels from surgical wound, the proportion of men whose HIV status is unknown who are HIV infected (Sub-study 1).***

Justification: HIV testing and counseling is one of the components of comprehensive VMMC services recommended by the World Health Organization and the Government of Kenya. However, our program data indicate that testing currently stands at about 74%, and questions arise if those who decline testing could have higher prevalence than those accepting to be tested. While VMMC is not denied to those who test positive for non-HIV prevention reasons, counseling messages for those known to be HIV infected differ from those who are HIV-negative. It is therefore important to compare HIV prevalence among those who opt to know their HIV status and those with unknown status to provide information that will inform counseling messaging among the latter group.

We will perform the testing of blood from bleeding vessels of those of unknown HIV status. The samples will be sent for testing at the KEMRI/CDC Laboratory in Kisumu.

***Aim 3: Determine the cost of providing VMMC among the three interventions relative to each other and to no intervention.***

Justification: Costing of new interventions help in making policy decisions regarding their viability for adoption. Our programs data have indicated that with sufficient demand for services, which we hope to create among older men through the proposed interventions, the cost of VMMC are expected to go down markedly. We will extract uptake data from procurement, stores and accounting records in terms of numbers of MCs done, and identify all expenses that went into each intervention and calculate the unit cost of each demand creation intervention.

## RESEARCH STRATEGY/APPROACH

## **STUDY DESIGN**

We propose to conduct a cluster randomized-controlled trial, with Locations forming the unit of randomization. Locations are the third smallest administrative units in Kenya after Province, District and Division and before Sub-Location and Village. Within the selected Locations, we shall randomly select approximately 16% of the villages which will be sufficient to generate the required sample size (see Tables 4 & 5 below). We shall visit 209 villages, as explained below (Table 5).

**Sample size Determination**

Sample size for detecting the impact of the interventions on VMMC uptake

Unpublished data from an on-going demand creation study and an HIV community based study show an average of 30% circumcision prevalence in the target group (25-39 years); a corresponding refusal rate of 15% and loss to follow-up of 15%. Using an average MC uptake of 30%, an intra-cluster correlation coefficient (ICC) of 0.005, and a design effect of 2.5 in sample size formula for cluster studies, we estimated that in order to be able demonstrate at least a 33.3 % increase in the rate of VMMC among young adult men in intervention arms when compared to those in control arm with 80% statistical power, and Bonferroni adjusted two-sided $\alpha=(\frac{0.05}{3})=0.017$ following the implementation of study interventions , we will need a sample size of 4,932 men reached with the intervention.

The graph below (*Figure 1)* shows that an effect size equal to 33.3% increase in MC from 30% to 40% will require about 1233 participants per study arm (total 4932), while an effect size equal to 50% increase in VMMC from 30% to 45% will require 573 participants per study arm (total 2292).

Adjusting for refusal rate of 15% and a loss to follow up of 15%, suggests contacting 1603 per study arm resulting in a total of 6412 uncircumcised men aged 25-39 years.

Fig.1 shows a range of sample size requirements for detecting various percentage increases (effect sizes) in VMMC uptake due to the interventions assuming causal effect, and for an 80% power, a 1.7% significance level and a two sided test.


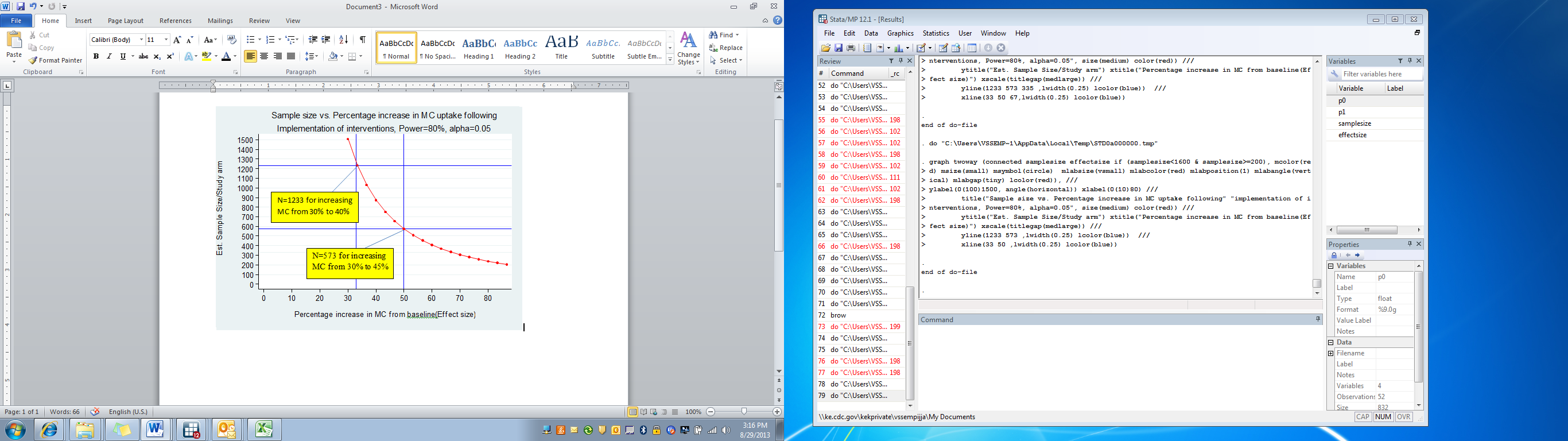


Figure 1: Sample Size Vs Percentage Increase in MC Uptake

Selection of Clusters (Locations):

The CONSORT (consolidated standards of reporting trials) extension for cluster randomized trials which provide guidelines on quality of reporting and study methodology of cluster randomized trials recommends a minimum of 4 clusters per arm.^[[24]](#endnote-24)^ Since greater statistical power and precision are obtained by sampling more clusters than sampling a higher number of study participants per cluster, we sought to sample as many eligible locations as was feasible as long as they did not share boundaries. Out of the 164 Locations across nine study districts^[[25]](#footnote-1)^ in Nyanza Province (Figure 1), we will systematically selected 45 Locations. Each Location is expected to have about 700 men in the 25-39 age bracket^[[26]](#endnote-25)^ of whom about 500 are likely to be uncircumcised (based on circumcision program data and demographic surveillance survey which is about 30%).

The selection and randomization of participating Locations was accomplished by a team of three (KA, EO and Dickens Omondi – a former study coordinator). The four steps below describe the process of selecting and randomizing the 45 Locations where the study will be conducted:

1. Sampling frame (total number of Locations in IRDO districts): According to the 2009 Kenya Population Census Report, Nyanza Province has 14 districts and 233 **administrative Locations** inhabited by traditionally non-circumcising *Luo* ethnic community (i.e. where circumcision is not a cultural practice). IRDO currently provides VMMC services in 12 of the 14 districts, and 194 of the 233 Locations. Out of these, the study will be done in 11 districts. Another study is planned for the one remaining district under IRDO. We contracted a cartographer to generate a GIS map showing the 11 districts and 164 Locations where IRDO will conduct this study (Fig 2: Map 1).
2. Separating eligible from ineligible Locations: To ensure the populations in participating Locations are as homogenous as possible in terms of ethnicity (which defines circumcising and non-circumcising communities), we spread the map on a flat surface and systematically identified and excluded 37 Locations: 34 bordering traditionally circumcising communities in Kuria, Gucha, Central Kisii, Nandi, Vihiga, Butere-Mumias and Busia districts, and 3 within cosmopolitan Kisumu city where mixed ethnic communities live. In addition, we excluded 5 Locations in the islands due to poor access. Consequently, we remained with 122 eligible Locations to select from (Fig 2: Map 2).
3. Selecting participating Locations: Of the remaining 122 eligible Locations, we randomly selected the starting point and went through the process of systematically selecting each Location and excluding all the contiguous ones until we identified the maximum possible Locations that did not share a boundary. This exercise was done repeatedly by randomly selecting different Locations as the starting point and seeing how many Locations we ended up with. The goal was to select the highest number while ensuring none is proximal to each other. Based on these selection criteria, 45 was the maximum number of Locations that could be included in the study (Fig. 2: Map 3).
4. Random assignment of participating Locations: The names of the 45 Locations were compiled and submitted to the study Biostatisticians (FO & VS) for randomization.The names were entered into Stata V12 statistical package and a randomization schedule produced using the random-number generator. Using simple randomization to allocate clusters, roughly equal number of Locations (11-12 per arm) was allocated to each of the four study arms (Fig 2: Map 4).

A summary of the steps and the number of locations are shown in *Table 1* below while randomization and distribution of allocation to the study arms by districts are shown in Tables 2 and 3.

**Table 1:** Summary of Selected Study Locations

| **Total Locations in *Luo* Nyanza Province** | 233 |
| --- | --- |
| Locations where IRDO is providing VMMC services | 194 |
| Locations excluded because of an ongoing pilot demand creation study | 30 |
| Locations excluded because they border traditionally-circumcising communities | 34 |
| Locations excluded because of having a multi-ethnic population | 3 |
| Locations excluded because of poor accessibility (Islands) | 5 |
| Locations eligible for selection | 122 |
| Locations excluded because they are proximal to a previously selected Location | 77 |
| Number of Locations selected for the study | 45 |

**Table 2:** The number of Locations assigned to each study arm by district

|  | **Study Arm** | | | | **Total** |
| --- | --- | --- | --- | --- | --- |
| **District** | **Control** | **IPC** | **DSO** | **IPC+DSO** |  |
| Bondo | 1 | 1 | 1 | 1 | 4 |
| Homabay | 1 | 1 | 2 | 1 | 5 |
| Ndhiwa | 1 | 1 | 2 | 1 | 5 |
| Kisumu East | 1 | 0 | 2 | 1 | 4 |
| Kisumu West | 1 | 1 | 0 | 1 | 3 |
| Nyatike | 1 | 2 | 1 | 2 | 6 |
| Migori | 1 | 0 | 0 | 1 | 2 |
| Rarieda | 1 | 1 | 0 | 0 | 2 |
| Rongo | 0 | 1 | 0 | 2 | 3 |
| Siaya | 1 | 2 | 1 | 2 | 6 |
| Suba | 2 | 1 | 2 | 0 | 5 |
| Totals | 11 | 11 | 11 | 12 | 45 |

***Map 1: All 11 Study Districts & Locations*** ***Map 2: Eligible & Excluded Locations***

***Map 3: 45 Selected Locations*** ***Map 4: The 4 Study Arms***

**Figure 2:** Maps 1-4 showing selection steps

**Table 3:** Randomization schedule for selected Locations

| **DISTRICT** | **LOCATION NAME** | **STUDY ARM** | **Study Region** |
| --- | --- | --- | --- |
| Ndhiwa | Central Kabuoch | IPC arm | 1 |
|  | Central Kanyamwa | Control arm |  |
|  | Central Kanyidoto | IPC and DSO arm |  |
|  | East Kwambwai | DSO arm |  |
|  | North Kanyamwa | DSO arm |  |
| Homa-Bay | Gem West | IPC arm |  |
|  | Homa Bay Township | Control arm |  |
|  | West Kochia | DSO arm |  |
|  | Gem East | DSO arm |  |
|  | Gongo | IPC and DSO arm |  |
| Suba | Gembe East | Control arm |  |
|  | Gwasi North | IPC arm |  |
|  | Gwasi South | DSO arm |  |
|  | Kaksingri East | Control arm |  |
|  | Rusinga West | DSO arm |  |
| Kisumu East | West Kolwa | DSO arm | 2 |
|  | Central Kisumu | DSO arm |  |
|  | East Kolwa | IPC and DSO arm |  |
|  | Kawino | Control arm |  |
| Kisumu West | N.C Seme | Control arm |  |
|  | North-West Kisumu | IPC arm |  |
|  | S.W. Seme | IPC and DSO arm |  |
| Migori | Lower Suna | IPC and DSO arm | 3 |
|  | Suna East | Control arm |  |
| Nyatike | East Karungu | IPC arm |  |
|  | Kaler | Control arm |  |
|  | West Muhuru | IPC and DSO arm |  |
|  | North Kadem | IPC arm |  |
|  | South East Muhuru | DSO arm |  |
|  | West Kadem | IPC and DSO arm |  |
| Rongo | West Sakwa | IPC and DSO arm |  |
|  | Lower Kanyamkago | IPC arm |  |
|  | West Kanyamkago | IPC and DSO arm |  |
| Bondo | Central Sakwa | DSO arm | 4 |
|  | East Yimbo | IPC arm |  |
|  | North Sakwa | Control arm |  |
|  | West Yimbo | IPC and DSO arm |  |
| Rarieda | South Asembo | Control arm |  |
|  | West Uyoma | IPC arm |  |
| Siaya | West Gem | IPC and DSO arm |  |
|  | West Uholo | IPC arm |  |
|  | N.West Gem | IPC and DSO arm |  |
|  | S.W. Alego | Control arm |  |
|  | Township | DSO arm |  |
|  | Ukwala | IPC arm |  |

1. Random selection of participating Villages from each of the 45 study Locations: According to the 2009 Kenya population census report, the selected 45 study Locations have a total of 165,514 households divided into 1,312 Villages. The three steps below describe the process of selecting representative Villages from each of the 45 Locations that will participate in the study:
   1. Since the census report does not provide population data by age at the Location level, we used the number of households (HH) and number of men in our target group (25-39 years) at the district level (2009 census) to calculate the average # of men aged 25-39 years per 100 HH (column 4 Table 4).
   2. Using the average # of estimated men aged 25-39 years/100 HH by districts in (a) above, we estimated the population of uncircumcised men aged 25-39 years in the 45 study Locations (column 3 table 5) and used this to calculate the # of men to be contacted per study Location (column 4 table 5) using probability proportion to population size.
   3. We then used the above information to estimate the # of HH to be visited (column 5 table 5), which allowed us to arrive at the number of participating Villages to be selected (column 6 table 5).

**Table 4:** Average # Men Aged 25-39/100 Households

| District | Population aged 25-39 | No of HHs | Average no of men (25-39)/100 HH | Average (men 25-39 yrs) non-circumcised /100 HH |
| --- | --- | --- | --- | --- |
| Bondo | 14288 | 37296 | 38 | 27 |
| Homa Bay/Ndhiwa | 25123 | 79540 | 32 | 22 |
| Kisumu East | 55666 | 115502 | 48 | 34 |
| Migori/Nyatike | 26648 | 70516 | 38 | 26 |
| Suba | 18769 | 45320 | 41 | 29 |
| Kisumu West | 9850 | 32992 | 30 | 21 |
| Rongo | 24937 | 67895 | 37 | 26 |
| Siaya | 36997 | 130705 | 28 | 20 |
| Rarieda | 9888 | 31033 | 32 | 22 |
| Total | 222166 | 610799 | - | - |
| Average | - | - | 36 | 25 |

**Table 5:** Estimated Target Population and # of Selected Villages

| **District** | **Location Name** | **# of Villages** | **Population uncircumcised aged 25-39 yrs** | **# of men to be contacted / Loc.** | **# of HH to be Visited** | **# Villages Selected** |
| --- | --- | --- | --- | --- | --- | --- |
| Ndhiwa | Central Kabuoch | 9 | 957 | 142 | 569 | 2 |
|  | Central Kanyamwa | 12 | 703 | 104 | 417 | 2 |
|  | Central Kanyidoto | 15 | 252 | 37 | 150 | 2 |
|  | East Kwambwai | 28 | 754 | 112 | 448 | 4 |
|  | North Kanyamwa | 16 | 435 | 65 | 259 | 3 |
| Homa-Bay | Gem West | 29 | 639 | 95 | 379 | 4 |
|  | Homa Bay Township | 40 | 1,957 | 291 | 1162 | 6 |
|  | West Kochia | 29 | 314 | 47 | 186 | 4 |
|  | Gem East | 15 | 567 | 84 | 337 | 2 |
|  | Gongo | 8 | 337 | 50 | 200 | 2 |
| Suba | Gembe East | 23 | 782 | 116 | 465 | 4 |
|  | Gwasi North | 42 | 926 | 138 | 550 | 8 |
|  | Gwasi South | 19 | 559 | 83 | 332 | 4 |
|  | Kaksingri East | 28 | 407 | 60 | 242 | 5 |
|  | Rusinga West | 32 | 815 | 121 | 484 | 6 |
| Kisumu East | West Kolwa | 18 | 8236 | 1223 | 4891 | 4 |
|  | Central Kisumu | 14 | 1272 | 189 | 755 | 3 |
|  | East Kolwa | 40 | 1551 | 230 | 921 | 8 |
|  | Kawino | 56 | 1097 | 160 | 641 | 12 |
| Kisumu West | N.C Seme | 47 | 763 | 113 | 453 | 6 |
|  | North-West Kisumu | 51 | 1051 | 156 | 624 | 12 |
|  | S.W. Seme | 69 | 799 | 119 | 475 | 5 |
| Migori | Lower Suna | 29 | 757 | 112 | 449 | 5 |
|  | Suna East | 24 | 455 | 68 | 270 | 4 |
| Nyatike | East Karungu | 23 | 288 | 43 | 171 | 4 |
|  | Kaler | 13 | 522 | 78 | 310 | 2 |
|  | West Muhuru | 10 | 250 | 37 | 148 | 2 |
|  | North Kadem | 19 | 471 | 70 | 280 | 3 |
|  | South East Muhuru | 16 | 165 | 24 | 98 | 3 |
|  | West Kadem | 17 | 797 | 118 | 474 | 3 |
| Rongo | West Sakwa | 49 | 1033 | 180 | 720 | 8 |
|  | Lower Kanyamkago | 33 | 1212 | 93 | 371 | 5 |
|  | West Kanyamkago | 31 | 624 | 207 | 826 | 5 |
| Bondo | Central Sakwa | 47 | 1391 | 237 | 948 | 8 |
|  | East Yimbo | 18 | 560 | 83 | 333 | 3 |
|  | North Sakwa | 21 | 567 | 84 | 337 | 4 |
|  | West Yimbo | 15 | 1469 | 218 | 872 | 3 |
| Rarieda | South Asembo | 28 | 537 | 80 | 319 | 4 |
|  | West Uyoma | 29 | 1029 | 153 | 611 | 4 |
| Siaya | West Gem | 22 | 308 | 46 | 183 | 3 |
|  | West Uholo | 39 | 848 | 126 | 504 | 5 |
|  | N.West Gem | 45 | 688 | 102 | 409 | 6 |
|  | S.W. Alego | 58 | 858 | 127 | 509 | 7 |
|  | Township | 36 | 1593 | 236 | 946 | 4 |
|  | Ukwala | 50 | 1030 | 153 | 612 | 6 |
| Total |  | **1,312** | **43,172** | 6412 | 25640 | 209 |

We will need to reach 25,640 households to contact 6412 uncircumcised men aged 25-39 years.

**Phases of Data Collection:** The study will be conducted in five phases, as follows:

Phase 1: Household Enumeration

Phase 2: Conducting of rapid formative research to confirm barriers and facilitators specific to older men, and to identifying intervention messages and preferred delivery days/times by this sub-population.

Phase 3: Establishment of baseline VMMC prevalence and the proportion of eligible men (uncircumcised, aged 25-39 years) in each participating village.

Phase 4: Implementation of the interventions.

Phase 5: Establishment of the endline VMMC uptake to estimate the impact of the interventions.

# PHASE 1: HOUSEHOLD ENUMERATION

To ascertain the size of eligible population (uncircumcised men aged 25-39 years), we will conduct complete household listing in all selected villages and enumerate male members in each household aged 25-39 years (Appendix 1: Household Enumeration Form). During this exercise, we shall physically visit all homes in the study villages and assign a unique identification number to every household (HIN ), which will be preceded by a pre-assigned study Location and village codes (e.g., KAW/KOD/1234 for Kawino Location and Kodongo village and Household number 1234). Enumeration shall be through home visits ONLY and not by any other means. The unique HIN assigned during the enumeration will be entered on Excel database and households with men aged 25-39 years will be sorted out and put on a separate list. The names and identifying information shall be kept separately from the main database (in a locked, password protected file) and will not be accessible to the person(s) working on the analysis.

Names of heads of household: We shall obtain names of heads of households during enumeration to help us identify selected households when we return for screening and enrollment. In this community, homes are identified using names of household heads. As such these names will be entered in the database. However, they will not be used for any analysis. Access to this data will be pass-word protected and restricted to the data manager and other authorized staff only. This information is strictly for purposes of locating the homes for the return visits to consent and enroll participants, to collect baseline and endline data, and to implement the intervention. Because of the expansive geographical coverage of study sites, we will organize coordination in 4 regions as in Table 3 above, based on proximity and logistics. Each Region will have a coordinator responsible for study activities on a day-to-day basis and reporting to the Study Coordinator. At the regional offices, we will have a data office for entry of data collected in the respective regions; the officer will be reporting to the data manager at the head office. Both at the study coordinating center in Kisumu and the regional offices, data will be entered in password-protected computers and stored in locked room accessible to data staff, regional coordinators, data manager and PIs. After data submission to the head office in Kisumu (using a password-protected flash drive), only the PIs, data manager, study statisticians and study coordinator will have access to the consolidated data base.

Because of the non-intrusive nature of the data to be collected, we shall obtain oral consent from the head of household or any adult member who can give us information on only the age and sex of household members. We therefore request for a waiver of documentation of written consent.

At this visit we will also collect names and telephone numbers (where known/available) of men aged 25-39 years to help us prepare a list of those to be reached by the intervention and contact them by phone or home visit to make appointments for baseline survey. We will also collect locator information of households with men aged 25-39 years, to guide the study staff who will return later for enrolment visit. At the end of the household enumeration, the data manager will prepare a complete listing of potentially eligible men in an excel sheet and the RAs will visit everyone in this list. During enrollment the RA will administer consent, assign Participant Unique ID (see pg xx), conduct baseline interview and verify the MC status of consented participants.

The list will be used to create a log of eligible men in each village. Determining the number of households with eligible men will help in knowing the denominator and in planning for recruitment. Once this information is obtained, we will use it to start planning for Phase 3 of the study (Conducting Rapid Formative Study). Phase 2 (Establishing Baseline VMMC Prevalence and Proportion of Eligible Men) of the study, described here below, will be implemented concurrently with Phase 1. In addition, we have drafted the tool [to be known as *VMMC Demand Creation Tool*] which will be used for demand creation in IPC and IPC+DSO Locations. Before being used, this tool will be updated with information collected from the formative phase of the study and submitted to the two IRBs for approval prior to implementation. We have attached herewith the draft version for initial review (Appendix 16: VMMC Demand Creation Tool). All eligible men in the selected villages will be approached to join the study.

# PHASE 2: CONDUCTING RAPID FORMATIVE STUDY (SUB-STUDY 1)

**Justification:** This phase is intended to provide information for use in delivering the interventions. We have already drafted messages based on existing literature for use in IPC and IPC+DSO intervention Locations/villages. However, since almost all the available information on barriers to and facilitators of VMMC in literature were collected among men of all ages and were not disaggregated by age group,^21,23^ we cannot assume they are automatically relevant to older men. As such, it will still be necessary to conduct a rapid formative study to explore reasons why older men aged 25-39 years go or do not go for VMMC services. Information obtained from this phase will be used to modify, if indicated, the messages developed from existing literature. We wish to emphasize that only systematic review and drafting of messages will be done while we await ethics approval for the messages, and certainly no data collection will be done before obtaining ethics approvals from both KNH-ERC and CDC IRB.

## SUB-STUDY DESIGN

This phase shall comprise of Focus Group Discussions (FGDs) and In-Depth Interviews [[4](#_ENREF_4)]. Twelve FGDs, with 6-10 participants, will be held with six groups of circumcised men and six groups of uncircumcised men, as follows:

- 1. 25-29 in southern Nyanza
  2. 30-34 in southern Nyanza
  3. 35-39 in southern Nyanza
  4. 25-29 in northern Nyanza
  5. 30-34 in northern Nyanza
  6. 35-39 in northern Nyanza

Similarly, twelve FGDs, with 6-10 participants, will be held with six groups of female partners of circumcised and six groups of female partners of uncircumcised men to obtain information to be used if men come with their female partners during the intervention or raise issues about women and VMMC. We will also use the FGD sessions to confirm the suitability and completeness of the messages we shall have developed from existing literature on demand creation.

Besides FGDs, we will conduct 48 IDIs to capture confidential information that some men and women would otherwise not divulge in an FGD setting. The IDI with male participants will comprise of four circumcised men and four uncircumcised men in each of the following age categories: 25-29 years, 30-34 years and 35-39 years. The IDIs with circumcised men and their partners will explore the same issues as the FGDs but with more specific questions about respondents’ experience at the circumcision site during service provision and sexual behavior post-MC (circumcised group, Appendix 9 & 12). For uncircumcised participants, specific questions will explore barriers within the families (e.g., with spouses, parents, children, siblings) or among friends and peers, as well as perceptions of the effect of VMMC on sexual performance (Appendix 10 & 13).

The IDIs with female participants will comprise of four partners of circumcised men and four partners uncircumcised men in each of the following age categories: 25-29 years, 30-34 years and 35-39 years.

We will also use the FGDs and IDIs to identify key and preferred sources of information on VMMC; which information type and source appeal to older men, which ones do not and why; and what messages convinced (if circumcised) or would convince (if uncircumcised) them and/or their peers to take up VMMC. To help in selecting appropriate people to serve as recruiters or service providers, we will collect their views on preferred characteristics (e.g., age, sex, etc). We will also obtain information on appropriate forums and strategies to reach older men with VMMC information one-on-one or in small group (e.g., work place, home, place of worship, etc).

The FGDs and IDIs will be conducted by trained study staff, with at least a diploma in social sciences or related field, and two years of research interviewing experience. They will be trained on ethics and protocol, and taken through intense practice with the guides until they become competent. The interviewers will be same gender as participants and fluent in English and the local language (*Dholuo*). Both FGDs and IDIs will take approximately one and a half hours.

**Inclusion/Exclusion Criteria**

***Focus Group Discussions and In-depth Interviews***

*Male participants:*

Inclusion:

- Aged 25-39 years
- Discloses status as circumcised or uncircumcised
- Gives consent to participate in the FGD and be audio-recorded.
- Resident of any of the 77 Locations outside main study area, within the 11 study districts.

Exclusion:

- Outside the 25-39 years age bracket
- Does not disclose circumcision status
- Non-resident of any of the 77 Locations outside main study area, within the 11 study districts.
- Declines to consent to participate in the study

*Female participants* [these are general women reporting having circumcised or uncircumcised partners, and not necessarily partners of men above]:

Inclusion:

- Aged ≥18 years
- Discloses that their partner’s circumcision status
- Gives consent to participate in the discussion and be audio-recorded.
- Resident of any of the 77 Locations outside main study area, within the 11 study districts.

Exclusion:

- Below 18 years of age
- Does not disclose circumcision status of partner
- Not resident of any of the 77 Locations outside main study area, within the 11 study districts.
- Declines to consent to participate in the study

### Selection of the FGD/IDI Locations and Participants

The formative study Locations will be selected as follows:

- The FGDs will be done in both southern and northern Nyanza regions. After systematic elimination by factors such as proximity to circumcising communities, multi-ethnic urban populations, and geographical inaccessibility, 122 administration Locations were potentially eligible for inclusion in the study; of these, 45 that were not adjacent to each other were selected, leaving out 77 Locations. The FGDs and IDIs will be conducted in some of the 77 Locations which were otherwise eligible but not selected for study due to geographical proximity to those selected. The process of selection will be done as follows:
  - 36 Locations (18 in Southern and 18 in Northern Nyanza) will be randomly selected out of the remaining 77 eligible Locations that were not selected for the implementation of the interventions.
  - 12 FGDs and 24 IDIs (divided equally between men and women) will be conducted in each of the regions - Southern and Northern Nyanza.
  - In each Location, various opinion leaders will be identified and asked to refer one eligible male and female participant for either IDI or FGD, until the required sample size is reached. The opinion leaders will include Chiefs, Assistant Chiefs, Village Elders, teachers, religious leaders, male group leaders, etc. This will ensure a variety of nominees.

### Consenting

We will obtain Written consents (Appendix 4: Written Informed Consent for FGD with Circumcised and Uncircumcised and Appendix 6: Written ICF for Main Study FGD with Partners of Circumcised and Uncircumcised Men) to conduct FGDs using standard guides (Appendix 5: FGD Guide for Circumcised and Uncircumcised Men and Appendix 7: FGD Guide for Partners of Circumcised and Uncircumcised Me). We will also obtain written informed consents (Appendix 8: Written ICF for IDI with Circumcised and Uncircumcised Men and Appendix 11: Written ICF for IDIs with Female Partners) to conduct IDIs with circumcised men (Appendix 9: In-Depth Interview Guide for Circumcised Men) and uncircumcised men (Appendix 10: In-Depth Interview Guide for Uncircumcised Men), partners of circumcised men & partners of circumcised men (Appendix 12: In-Depth Interview Guide for Female Partners of circumcised Men) and partners of uncircumcised men (Appendix 13: In-Depth Interview Guide for Female Partners of Uncircumcised Men). Recruitment of participants and their composition are described in detail above. The study will be done to confirm barriers to and facilitators of VMMC uptake relevant to older men, from the perspective of both men and women. In addition, this phase will serve to identify appropriate VMMC messages and delivery forums for the target age group.

### Procedure of the FGD

The FGDs will be conducted with both circumcised and uncircumcised men. There shall be a moderator and the note taker who will document observations during the discussions. The group shall consist of 6 – 10 participants and will use the local language (*Dholuo*). There shall be an instruction manual attached to the questionnaire guide for the moderator to use.

Before starting off, the research assistant will:

1. Introduce self and clearly explain that this discussion is part of a research study on voluntary medical male circumcision (VMMC) to better understand what would make more men aged 25 to 39 years go for VMMC and that the discussion would take approximately 1½ hours;
2. Obtain a individual written consent and brief demographic information as participants come in;
3. Assign each participant a serial number for use when contributing to the discussion during the FGD, and issue number tags. Explain that this is necessary to make it easy for the researchers to follow the views contributed by each person while keeping personal identity confidential. Urge participants to not reveal names and personal details during the session;
4. Explain that the discussion will be audio-recorded to capture all contributions from participants, that audio tapes will be destroyed (digital recorders erased) after transcription (by the study coordinator and witnessed by the data manager) and that the recordings will not be used for any other purposes. Emphasize need to not mention name while recording is taking place;
5. Emphasize that taking part in the group discussion is voluntary and those not willing to be audio-taped are ineligible;
6. Confirm the audio-recorder is functioning, and switch it on for the discussion to start.

Data from the FGDs and IDIs will be transcribed, typed, coded and analyzed to extract information on top barriers to and facilitators of VMMC among older men. The results will be reviewed for concurrence with existing literature by the two PIs, together with the Nyanza-based co-investigators. The draft *VMMC Demand Creation Information Sheet for Older Men* developed with information from previous studies will be updated to incorporate any new information from the Formative Phase, **and submitted to KNH-ERC and CDC IRB for review and approval prior to implementation**.

While the recruitment message document (Appendix 16: *Toolkit for Addressing Barriers and Facilitators to VMMC*) will be undergoing ethical review, we will be setting up for the implementation of Phase 3 of the study.

# PHASE 3: ESTABLISHING BASELINE VMMC PREVALENCE AND PROPORTION OF ELIGIBLE MEN

Based on the eligible participants’ list, research assistants will only go back to households with men aged 25 to 39 years. Before administering enrolment consent, RAs will ask participants to identify a private location within or outside the house where they can do consenting and interviewing. The RA will then administer consent for participating in the study, enroll consenting participants (Appendix 2A: Written ICF for Enrollment and Verification of MC Status) and assign Participant Unique ID number. The number will comprise of the participant’s Location and Village codes, the first initials of the participant’s 3 names and a six digit number, preferably the last six digits of the participant’s national ID or telephone number, or another number selected by the participant if either of the national ID or telephone number is unavailable. These components will form the Participant Unique ID number. For example, Edward Albert Sungu from Kawino Location, Kodongo Village with 123456 as his six digit number, will have a Participant Unique ID number that will look like this: KAW/KOD/ E12/A34/S56.

At enrollment, participants will be given 3 months within which to go for circumcision, Participants can go whenever they want for VMMC, but our study will only capture their data if they go within 3 months. They will also be informed that a reminder of the expiry date for circumcision will be sent through text message to all participants one month before the expiry of the circumcision window period. The message will read: “***One month remaining. Take action for your health now***”. Participants will be informed at enrollment that they will be sent a text with the 3 words as a reminder that in case they have not gone for circumcision, they have 1 month remaining. This information has been captured in Written Informed Consent Form for Enrollment and Verification of Circumcision Status (Appendix 2A) as follows: **You have up to 3 months within which to go for circumcision. However, you are encouraged to go for circumcision as soon as possible; we recommend that it be within the next 3 months. Two months from now, you will be reminded through a text message to go for circumcision. The message will read: “*One month remaining.*** ***Take action for your health now*”.** The message is general and safe to receive even through a friend’s phone for those who do not own phones. A reminder is important because in an ongoing study on demand creation for VMMC in Kenya (funded by the Bill and Melinda Gates Foundation), preliminary results from the qualitative component reveal that participants who did not go for circumcision wished they were reminded because they simply forgot.

After assigning the participant unique ID number, the RA will conduct the baseline interview (Appendix 2B: 2 Questions for All Clients aged 25-39 yrs at Counseling), verify the MC status and inform participants of their intervention arm and deliver the intervention if participant is available (if not, another visit will be made at a later date to deliver the intervention). During the interview, we will document participants’ circumcision status from verbal report. After the interview, participants who consented to physical examination will be asked to choose where this can be performed. The identified location shall be acceptable to both the participant and RA. Physical verification will be done to minimize mis-reporting and/or misclassification, especially by those who are partially circumcised or who were born with short foreskins. Circumcision status will be recorded as fully circumcised (no foreskin), partially circumcised (foreskin is past corona sulcus but covers less than one half of the glans, in a flaccid state), and fully uncircumcised (foreskin covers half or more of the glans, in a flaccid state) and recorded on Appendix 2B.

We estimate that consenting will take about 30 minutes, the interview will take about 45 minutes, penile inspection will take about 5 minutes, altogether approximately 1½ hours. Where the participant is ready for the intervention, the RA will proceed to provide the intervention given that the participant will have consented to it. The delivery of the IPC and IPC/DSO intervention will take additional 1 hour while in DSO-only Locations; the process will take additional 30 minutes. Where a participant does not have time, another visit will be made (see Phase 4 below).

Details of participants enrolled in the study will be entered into an excel Master Log (Appendix 14) which will contain all participant details; 3 names, ID number, age, location, village, telephone number of the participants or of close friend and date of enrollment.

**Inclusion/Exclusion Criteria for Baseline Phase**

Inclusion:

- Aged 25 – 39 years
- Resident of the study village - has been living in the physical structure identified during the enumeration and who has been eating together in the same household.
- Intends to continue living in the village for the next 9 months (which is the estimated duration of data collection).
- Gives written consent to participate in the study and the interventions.

Exclusion:

- Outside the age range of 25-39 years
- Non-resident of study villages
- Intends to relocate from the study village within the next 9 months
- Declines *to consent to participate in the study or the interventions*

**Maintaining confidentiality**:

The RAs enrolling participants in the households will have access to the participant unique ID number because they will be responsible for assigning the numbers. However, the RAs at the clinics where participants go for circumcision will not have access to the Master Log; instead they will call the Data Officer in charge the Master Log to confirm participant’s identity. Data Clerks will also have access to the participant unique ID number during data entry. All data will be collected by trained staff and kept under lock and key, accessed by designated study staff only. As described on page 20, under Phase 1: Household Enumeration, the components of the Participant Unique ID number have been systematically mixed in order to maintain participants’ privacy.

Once they complete their roles, household RAs assigning the participant unique ID numbers and Data Clerks entering the information on the data base will not have further access to participants’ personal information. Clinic-based RAs who will be confirming identity of participants will not have access to the Master Log – they will verify identity of participants by calling the Data Officer in-charge of the Master Log (Appendix 14) at the head office.

# PHASE 4: IMPLEMENTING THE INTERVENTIONS

The study has four intervention arms: Inter-Personal Communications (IPC), Dedicated Service Outlets (DSOs), a combination of IPC and DSO (IPC+DSO), and Control. We have described here below how each of the interventions will be implemented.

**Study Arm 1: Inter-personal communications (IPC) intervention:** We will advertise for interested behavioral counselors, clients from our VMMC program, community health workers, and other community recruiters (e.g. female partners, circumcised men), to be trained as VMMC interpersonal communicators (aka Research Assistants - RAs). We will add on to the list other preferred recruiters suggested by participants during the Formative Phase (sub-study 1). They will be trained for 5-6 days on the following contents: highlights of the observational studies prior to the MC clinical trials, the clinical trials design and results, how VMMC works to reduce HIV, operational research alongside VMMC rollout, and VMMC uptake in Kenya disaggregated by age. These will equip them with sufficient background information necessary to answer a wide array of questions that may arise during implementation of the interventions.

They will then be thoroughly trained on the barriers to and facilitators of VMMC among older men using information from previous studies as well as from the Formative Research phases of the study. The RAs will also be trained on and practice intervention implementation – how to approach potential participants in their households, how to deliver the messages, how to answer questions, and so on. After obtaining IRB approval, the trainees will participate in practical exercise where they will go out and put into practice the skills and share their experiences in a report-back session. This practical exercise will be in the form of role plays so that RAs get to practice with colleagues what they have learnt during training. A pre-post training evaluation exercise, as well as performance during practicals, will be used to assess competency and identify those qualified to be engaged as recruiters.

Recruitment Approach: Depending on participant’s availability, this phase can be conducted together with Phase 2 if the intervention tool (Appendix 6) has been approved, and done subsequent to verification of MC status; alternatively, another visit will be made if participants do not have time or if Appendix 6 has not been approved. If another visit is needed, we will visit every household with enrolled participants at a time when they are likely to be at home (with their spouses where applicable) to discuss VMMC using the demand creation tool (Appendix 16: *VMMC Demand Creation Tool kit*) as described in the next sub-section.

Participants in IPC or IPC/DSO Location will receive information on VMMC while those in DSO Locations will be informed about, and given directions to VMMC sites designated to older men. During consenting, participants will be informed that by consenting to be enrolled in the study, they are consenting to be assigned into one of the three intervention arms or control. Hence, by consenting to join the study they will also have consented to participating in the respective interventions. After delivering the intervention (whether at enrollment visit or a subsequent one), participants will be given a pre-designed referral coupon (Appendix 15: Referral Coupon) to take along when going for services. They will also be asked to memorize the serial number if possible. The RAs will be expected to return to the same households up to three times (within a period of three month) to reach those not found at home during the previous visit – this applies to both enrollment visit and the visit to deliver the intervention, if different from enrollment visit.

Delivery Process of IPC Intervention: We propose an approach whereby a trained RA (behavioral counselors, circumcised men, CHWs, or any other cadre of individuals identified during the formative phase(e.g. female partners, circumcised men) will meet older men in their households, and do the following: i) approach the person(s), introduce self as an RA from IRDO, develop rapport and request for time to discuss briefly about VMMC; ii) strike a conversation on reasons why they or their peers have not gone to be circumcised as well as reasons that would make them get circumcised, jotting down responses to guide the discussion. The responses will be written down in a notebook with carbonated duplicate pages per participant (**APPENDIX 44: DOCUMENTING DISCUSSION AT IPC AND IPC/DSO LOCATIONS**). The top page will be submitted for data entry and the copy will remain in the notebook. Only the Participant’s ID# (described on pg. 25), District, Location, Village and RA ID # will be indicated; iii) using the *Toolkit for Addressing Barriers and Facilitators to VMMC (Appendix 16)*, address each reported barrier carefully and completely, always ensuring the person(s) is/are engaged in the discussion (the goal is to pass correct and complete information in a relaxed and conversational manner); iv) revisiting each stated barrier and exploring what they now think about them after the discussion, explore whether the same barriers would still deter them/their peers from getting circumcised, and address any new or lingering concerns.

“Data collected from this exercise will first be coded by key themes, such as myths, misconceptions or misunderstandings. For each theme, we will obtain frequencies of mention; for example, for each misunderstanding, wrong length of time for sexual abstinence, wrong number of pain injections administered, wrong understanding of where injection is given or what gets surgically removed, etc. will be tallied. We will then document the extent of each misunderstanding by reporting the frequency and proportion of mention. We will link the Participant ID# to get his demographic data (age, education, marital status, and employment status) from **Appendix 2B: ENROLLMENT QUESTIONNAIRE AND CIRCUMCISION VERIFICATION AT BASELINE AND ENDLINE**. No personal identifiers will be collected or reported specifically for this activity.”

Moving on to the facilitators for VMMC, the RA will then pick each benefit/facilitator mentioned by the person/family members and reiterate their importance, ensuring sufficient and correct information is given. To conclude, the RA will encourage the men to seek VMMC and ask them to urge their family members and friends to also go for circumcision. The RA will leave behind a referral coupon (Appendix 15) indicating sites where VMMC is being offered in the neighborhood and contact information of team leaders in case of questions. Copies of *Appendix 17: ‘All You Need to Know About VMMC’ booklet* will be left with each person/family reached, for ongoing reference and to share with others.

While different RAs will reach out to potential participants to create demand for VMMC and make referrals to service provision sites, the effectiveness of each cadre of RA (behavioral counselors, CHWs, teachers, VMMC clients, etc) will be assessed through the number of referral coupons surrendered at the VMMC clinic after circumcision. Those who have forgotten/lost their referral coupons will be asked for the serial number of their referral coupon, which will be confirmed from the Tracking Log (Appendix 3A) and matched with the RA assigned to the respective Location and village.

**Study Arm 2: Designated service outlets (DSO) intervention** One of the concerns older men have expressed about going for VMMC services at current facilities is the fact that they are made to queue with age mates of their children.^23^ They find this embarrassing given VMMC is a private affair and one that touches on sexuality, an often taboo subject between parent and child in many African households.^26^ Also, because VMMC has been largely marketed as an HIV and STI prevention strategy, older men believe young people may perceive them as going for circumcision because they are promiscuous. To make older men feel comfortable going for VMMC, we plan to set up DSOs to serve men aged ≥25 years, exclusively.

Recruitment Approach and Delivery of the DSO Intervention: If not done at enrollment visit, the RAs shall visit every household with eligible men (uncircumcised, age 25-39 years) in Arm 2 to inform them about the availability of DSO sites in their neighborhoods. For participants in this arm, the RAs will describe DSOs as sites where services are offered exclusively to men aged ≥25 years. These will include designated health facilities (including private facilities) and designated days of the week in selected health facilities. They will also include providing services in the evenings and weekends, and through special mobile services targeting older men only. In all the DSO sites, participants will be served by male staff in the same age bracket.

Potential participants will also be informed that at DSO sites we will strive to shorten the waiting time to no more than three hours. A floating circumcision team will be called upon whenever there is a surge of participants, and we shall establish a system of rebooking participants who arrive late, or as soon as it is apparent that surgery cannot be done the same day. There will also be information that all other VMMC sites continue to serve all men regardless of age (i.e., including older men) while DSO sites will only serve men aged ≥25 years.

After describing the characteristics of DSO sites, the RA will share - both verbally and through a flyer (Appendix 18: DSO Sites and Location Information) - information on where to find DSO sites in the respective Location. If there are questions from participants, they will respond using: *All You Need to Know About VMMC* booklet (Appendix 17), the same way current recruiters do.

To monitor which questions are asked and how they are responded to, we have developed a monitoring tool (Appendix 19: Monitoring Form for Demand Creation Strategies) to document demand creation activities, and have included questions asked by participants. Staff will be trained on how to pass sufficient information using Appendix 17 without going into details contained in IPC. The monitoring tool will enable us to identify DSO and Control participants who may have received information that puts them close to IPC recipients. In addition, different group of RAs will be trained only on the intervention they will deliver, thus minimizing the chance that DSO RAs will have sufficient information on IPC to conflate the two during intervention delivery.

**Study Arm 3: Combined IPC and DSO interventions:** In selected Locations, IPC and DSO interventions (as described above) will be implemented concurrently. This will be done to determine the effect of both interventions delivered jointly compared to each delivered singly, and compared to no intervention.

Delivery Process of IPC+DSO Intervention: Activities and processes described under IPC and DSO will be replicated in these Locations/villages. The RAs will inform their participants about DSO sites and non-DSO sites in their neighborhoods, to allow people make a choice on where to go. Participants will be informed that services continue to be offered at all other facilities regardless of age. Copies of *Appendix 17: ‘All You Need to Know About VMMC’ booklet* will be left with each person/family reached in IPC+DSO sites, for ongoing reference and to share with others.

Participants aged 25-39 years in DSO and DSO+IPC Locations seeking services in non-DSO sites will be asked question 2 in Appendix 20 B (*What or who influenced your decision to come to this site while there are VMMC sites dedicated to men your age in this Location?*). The information will be useful in understanding reasons for preference for non-DSO sites yet DSO sites are available.

**Study Arm 4: Control/No Intervention:** One-quarter of participating Locations has been randomly assigned to the Control or No Intervention or ‘standard of care’ Arm. In these Locations, participants will only be reached with information on VMMC with reference to the ’*All You Need to Know About VMMC’* booklet (Appendix 17) at the time of enrollment.

### Demand creation for all other ages

Because the study targets only older men, demand creation for younger men/boys will continue as part of program work. As such, routine recruitment activities will continue for all men in all Locations; these include distributing information, education and communication materials; conducting road shows and magnet theater events to promote VMMC services; carrying out one-on-one or small group face to face interactions with community members, asking those who have been circumcised to refer others; visiting social events to pass information and invite men for service; using radio to present information of VMMC; and so on.

##

### Participant Inclusion and Exclusion Criteria

Inclusion criteria: We will include in the study men who are:

- Uncircumcised
- Aged 25 – 39 years
- Residents of the study village (has been living in the physical structure such as a compound or homestead identified during the enumeration and who has been consuming or making some contribution to food and other shared household resources).
- Intend to continue living in the village for the next 9 months, which is the estimated duration of data collection.
- Give written consent to participate in the study and the interventions.

During intervention, eligible participants will be asked to go for services with their National ID cards (if available), as well as with the referral coupon which will indicate district, Location and village of residence. Additionally, we will record participants’ three names, and last six digits of personal or friend’s telephone number. These will help in correctly identifying those who misplace their referral coupons (process described further on pg. 30). Participants who do not have a phone will be asked to give the phone number of a friend. The phone numbers and ID #s will be sorted immediately after data entry to ensure duplicates are identified, flagged such that when these participants come for services, they will be asked to describe the process of enrollment in the study to confirm they were enrolled. The RAs will ensure they at the very least mention being consented, interviewed, and asked to be physically examined to verify MC status.

Exclusion criteria: We will exclude from the study those who are;

- Circumcised
- Aged <25- or >39 years
- Non-resident of target villages
- Plans to move away from the village within nine months after enrolment
- Does not give consent to participate in the study

In addition, participants who misplace the referral card, have forgotten their Unique PIN and cannot be matched through their three names, date of birth (or age), village or phone number (matching process describe on pg. 30) will be excluded. Those who do not meet the eligibility criteria will be offered services but not included in the study.

Note: The study design and implementation is summarized in the flow chart, Fig. 3 below.


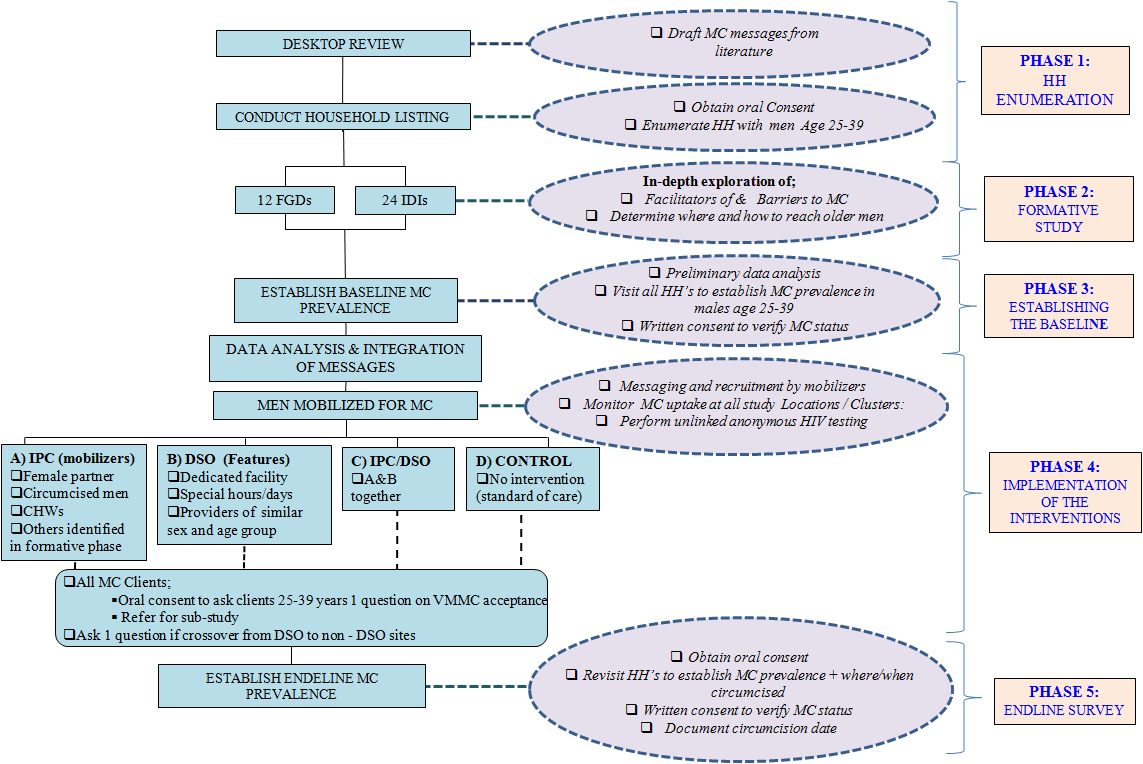


Figure 3: Schematic Presentation of study design and implementation

###

### Activities that cut across study arms:

1. Intervention Delivery
   1. In all the study Locations all participants aged 25-39 years coming for circumcision will be informed about the sub-study 4 (Role of sexual partners in time to resumption of sex post-circumcision) and given informational flyer (Appendix 31).
   2. We will continue to monitor recruitment activities in all arms to document other VMMC recruitment approaches that may be provided through other channels and which may bias the results.
   3. At all study sites, comprehensive VMMC services will be provided routinely per Ministry of Health guidelines, which includes: VMMC education and risk reduction counseling, HIV testing and counseling, condom education and distribution, STI screening and treatment, and the surgical procedure.
   4. At the end of the counseling session all participants aged 25-39 years accessing VMMC services at any of our study sites will be taken through written consent process and asked one question on what/who influenced their decision to come for VMMC (Appendix 20 A: Written Consent Form with 2 Questions for MC Participants Aged 25-39 Years). The question will help to identify which messages / cadre of RA / approaches are effective in influencing decision-making for VMMC for older men. In addition, men in DSO locations going for services in non-DSO sites will be asked question 2 from the same tool (Appendix 20 B). This will help us to understand why, despite availability of DSO sites, some older men still choose to go to non-DSO sites. Information from the two questions will not be linked to the respondents; instead, the questions and possible answers will be pre-printed on a piece of paper on which staff will simply check a participant’s response and drop in a box for collection by data staff. We will run descriptive statistics to determine top actual facilitators for circumcision.
2. Monitoring of Demand Creation Activities across Study Arms

We have designed a monitoring tool (Appendix 19: Monitoring Form for Demand Creation Strategies) to document information at different stages of the study; the form will capture:

1. Questions asked by participants during delivery of the intervention
2. Any additional information given beyond what is contained in Appendix 17: ‘*All You Need to Know About VMMC*’ booklet) in DSO and Control Arms.
3. Routine recruitment for the VMMC program in all study Arms to document if similar strategies were used during the study period.
4. Matching of participants enrolled in their houses with those coming for MC services:

It is important to track participants from the point of recruitment/referral to the VMMC site. We have designed a participant unique numbering system (see also pg 24 above) which makes it possible to track participants from point of referral to the VMMC site while minimizing disclosure of their identity, as described below:

- Every participant’s referral coupon will bear a pre-assigned study code that designates: Location (e.g., KAW), Village (e.g., KOD), participant’s 3 initials and an easy-to-remember six digit number which can be the last 6 digits of participant’s National ID or last six digits of his phone # or a friend’s phone # or any number (e.g. 123456). The complete code for participant Edward Albert Sungu from Kawino Location, Kodongo Village with # 123456, will therefore be: KAW/KOD/E12/A34/S56. The numbering in the Household listing form, the Tracking Log and the Referral coupons (Appendix 1, Appendix 3A and Appendix 15), will be synchronized with this numbering system to make it easier to track participants and link them to the intervention.

Additionally, the Tracking Log will also have the three names of the participant and his village, to be used in case he forgets to come with the referral coupon or does not recall the number. Thus, when a participant comes to the VMMC service site, they will be identified as follows:

1. Participants who have the referral coupon:
   - The Clinic-based RA will ask for the referral coupon (Appendix 15) given at enrolment.
   - S/he will then telephone the Data Officer based at the head office in Kisumu who maintains the Master Log in Excel (Appendix 14) and read out to the Data Officer the participant unique ID number.
   - The Data Officer will locate the Participant Unique ID number from the Master Log and tell the Clinic-based RA the three names, age, village and enrollment date of the participant, one at a time, as the clinic RA confirms with the participant.
   - If the details provided by the Data Officer match what the participant says, then he will be deemed correctly matched. Those not correctly matched will just receive circumcision services but will not be included in the study.
2. Participants who do not have the referral coupon:
   - The participant will be asked to show his national ID that should have the same number and three names recorded at enrollment. The national ID photo should resemble him.
   - The Clinic-based RA will then telephone the Data Officer based at the head office in Kisumu and read out the participant national ID number.
   - The Data Officer will confirm if the national ID number is in the Master Log. If it exists, he will then read out to the Clinic-based RA the three names, age, village and participant unique ID number, one at a time, as the clinic RA confirms with the participant.
   - If any two of the details provided by the Data Officer match what the participant says, he will be deemed correctly matched. Those not correctly matched will just receive circumcision services but will not be included in the study.
3. Participants who do not have both referral coupon and national ID:
   - The participant will be asked if he can remember his national ID number or the cell phone number he gave during enrollment.
   - The Clinic-based RA will then telephone the Data Officer based at the head office in Kisumu and read out the participant national ID number or cell phone number.
   - The Data Officer will confirm if the national ID number or cell phone number is in the Master Log. If either exists, he will then read out to the Clinic-based RA the three names, age, village and participant unique ID number, one at a time, as the clinic-based RA confirms with the participant.
   - If any three of the details provided by the Data Officer matches what the participant says, he will be deemed correctly matched.
   - If there is no match, the participant will just receive circumcision services but will not be included in the study.
4. Participants who do not bring referral coupon or national ID number and do not remember their unique ID number, national ID number or cell phone number:
   - The participant will be asked for his three names, preferably in the order in which he gave them during enrollment.
   - The Clinic-based RA will then telephone the Data Officer based at the head office in Kisumu and give the three names of the participant.
   - The Data Officer will search the names from the Master Log. Once found, the Clinic-based RA will ask the participant his age, village and Location. If these details match, the clinic-based RA will then ask the participant his reasons for coming to the facility. His response will reveal if the participant’s village was part of the location randomized to one of the intervention arms, and the self-reported reason(s) for coming to the site may indicate why they came (e.g., “I was told that there were special sites for older men here, so I came here” or “A person came to my house and talked to me about VMMC” etc.). During analysis, we may be able see if there are any differences by those who came with the coupon or any other information above vs. those who self-reported. If he fails to give reasons for coming and fails to describe any steps during enrolment, the participant will just receive circumcision services but will not be included in the study).

Since the Tracking Log will have identifier information (including 3 names, truncated ID number, truncated phone number, etc), we shall ensure confidentiality by locking up the log in a secure cabinet at the data office in the regional office and it will be accessible only to authorized staff, who will have signed the confidentiality agreement.

**Note**: There will be ONLY one Master Log kept at the head office in Kisumu with access limited to the Data Manager, Study Coordinator, Data Officer, 2 Statisticians and the PIs

#

# HIV TESTING OF PARTICIPANTS WITH UNKNOWN HIV STATUS (SUB-STUDY 1)

In our VMMC program and in other Kenyan programs as well, between 10% and 30% of men decline HIV testing. Given that VMMC is offered principally as an HIV prevention strategy, it is important to determine the HIV status of those who decline testing, and if the prevalence is significantly higher than those who accept testing, there will be need to design approaches to improve uptake of testing. This is particularly important following results from a recently concluded study that indicated that 44% of HIV-infected men resumed sex before the recommended 6 weeks of sexual abstinence^[[27]](#endnote-26)^.We will identify this group from the MOH VMMC Client Form, Version 2, May 2011 (Appendix 23: MoH VMMC Client Form). According to the MOH guidelines, anyone who is not tested on site and does not report being HIV+ is assumed to have unknown HIV status. This will be our population of interest, and will be identified if under Section B of the client form, item B1, question 2 (HIV status by self report) “Unknown” or “HIV” box is checked. While the section of the form is filled by the Counselor performing the test, the document moves with the client through subsequent stages and will be reviewed by the clinical staff performing physical exam (who will administer the written consent, Appendix 24 Written ICF for Unlinked HIV Testing on Blood Sample collected from Bleeding Vessels of Participants with Unknown HIV Status) as well as those performing the surgery (who will collect the blood and therefore need to identify eligible participants).

Sample size for HIV prevalence among uncircumcised men whose HIV status is unknown (*Sub-study 2*)

Currently in Kenya, there are no anonymous and/or unlinked HIV prevalence studies that evaluate the rates of HIV among uncircumcised men whose HIV status is unknown which can inform sample size estimation. We are also unaware of similar studies in other parts of Africa. However, studies from developed countries suggest that those whose HIV status is unknown may be at greater risk for HIV infection than those who accept testing.^[[28]](#endnote-27),^^[[29]](#endnote-28)^ Recently, researchers in Washington D.C, USA, tested de-identified blood samples from decliners of HIV testing. They found that the rate of HIV infection was nearly three-times higher in those who declined routine ED HIV testing compared with those who accept such testing.^38^ A similar finding had earlier been found in Atlanta-USA where patients who declined testing were three times more likely to be HIV infected than those patients who were tested.^39^ In Luxemburg-Netherlands, heterosexuals with a history of STI, current STI-related complaints, and those who had been warned that their partners were diagnosed with STI were more than two times as likely to refuse HIV testing.^[[30]](#endnote-29)^

Data from our VMMC programs that target older men in most (12/14) of the districts of Nyanza province inhabited by non-circumcising Luo community suggest that the prevalence of HIV among men taking up VMMC and who accept testing is 10.8%. These data also suggest that 23.6% of the men in the target age-group who accept VMMC decline HIV-testing. To be conservative, we assumed that the prevalence of HIV in this group of men is around two times that of similar men who accept such testing, translating into 21.6% HIV prevalence among uncircumcised men whose HIV status is unknown. If our assumption of twice the prevalence is wrong, we believe we shall have erred on the side of safety - we shall have erred on the side of over-estimating rather than underestimating the required sample size thus ending up with a bigger sample size than would have needed. However, we will benefit immensely in terms of precision of the study estimates. Using a design effect of 2.5 due to clustering, in the sample size for estimating prevalence^41^, we estimated that we will need to test randomly selected unlinked blood samples from 163 men per arm whose HIV status is unknown in order to estimate a HIV prevalence that falls within 10% points of the true population prevalence with 95% confidence. Our study staff will collect samples from all of men who refuse testing but consent to Unlinked HIV testing. Binkin et al. (1992)^41^ found that for a relatively common disease (as is HIV in the study area), if the desired level of precision is around ±10% points or less and the design effect is around 2, then collecting data from more than 30 clusters provides good estimates of the true (unknown) population parameters with the desired level of precision. Our study will be conducted in 45 clusters.

With a refusal rate of 23.6% voluntary HIV testing, and a consenting rate of 60%, then the rate of getting a man who refuses testing but consents to the testing of unlinked blood sample from his bleeding vessels is 14.2%.   In other words, of the 4932 participants in the main study we expect 1164 (4932*0.236) to refuse HIV testing. With assumed unlinked HIV testing acceptance rate of 60%, we can obtain 700 such men from our sample size of 4932 in the main study. For a design effect of 2.5, using a sample size of 700 for the sub-study will enable us to estimate the HIV prevalence of 21.6% with a precision of  4.8%=sqrt[(2.5*1.96^2*.216*.784)/700].

## Sub-study 2 Inclusion/Exclusion Criteria

Inclusion*:*

- Be a male aged 25-39
- Resident of one of the 45 study Locations
- Already enrolled in TASCO study (Main study)
- Has declined HIV testing
- Has consented to unlinked testing of his blood from the bleeding vessels/finger prick
- Sought services at a participating VMMC clinic.

Exclusion*:*

- Males outside the 25-39 age bracket
- Non-resident of one of the 45 study Locations
- Not enrolled in TASCO study
- Has accepted linked HIV testing
- Has not consented to unlinked testing of his blood from the bleeding vessels/finger prick.

## Enrollment of Sub-study Participants:

Participants for sub-study 3 will be enrolled from all the 45 Locations; however the Locations will be randomly assigned into nine (9) groups each with five clusters. Leaving the first two weeks to allow for the study set up and, have it up and running smoothly, and the last month for close out, enrollment into the study will take place over the remaining four and a half months (18 weeks). The first group will be assigned the first two weeks (i.e. week 3 & 4) to enroll the first 16 consenting participants; the next group of five clusters will be assigned the next two weeks (i.e. week 5 & 6) to enroll their participants and the process repeated until all the 45 clusters have been assigned their two-week enrollment period (ending week 19 & 20). After this period, enrollment will be opened for a further two weeks to all the clusters who will not have achieved the enrollment target. We believe that since each Location will be enrolling only 16 participants, the proposed arrangement allows for adequate time to enroll all the sub-study 3 participants and for enrolment to spread throughout the data collection period.

## Methods/Procedure:

We will perform unlinked testing of blood from bleeding vessels of those whose HIV status is unknown. While the call for proposals recommended testing blood from the excised foreskin, we are concerned that we may not be able to harvest sufficient blood from most foreskins to perform the test. This is because the forceps-guided method being used in Kenya^[[31]](#endnote-30)^ crushes the foreskin and compresses the blood vessels that supply the distal section. This results in little or no bleeding on the excised foreskin. We therefore propose to only collect blood samples from the bleeding vessels from participants (n=700).

We will also offer participants in this sub-study, the option of collection their Dried Blood Spot (DBS) blood samples from a finger prick. For participants who consent to unlinked HIV testing from bleeding vessels, the DBS sample will be collected during surgery. For participants who consent to unlinked HIV testing from finger pricking, the sample will be taken after surgery is completed but while the participant is still in the theatre. The surgeons and their assistants will be trained on both methods of blood collection (Dried Blood Spot and finger prick). The finger prick sample will be for DBS test and not be for rapid HIV test.

- - - 1. DBS Sample from Bleeding Vessels:

In a circumcision procedure, there are typically about 5 – 6 bleeding vessels, a result of the severance of the superficial vessels supplying the distal portions of the foreskin. Bleeding (generally minimal with good control) is usually stopped by ligation or cauterization. Immediately after excision of the foreskin, the raw area is exposed and the process of hemostasis is started. This is usually done by application of pressure around the raw area using dry and sterile gauze and helps to close up capillaries that may cause oozing of blood. This is followed by identification and clumping of individual bleeding vessels using haemostatic artery forceps. This step is usually done by the surgeon with the help of an assistant surgeon whose role is to swab and clear the surgical field to aid in the identification of the blood vessels. During this stage, the assistant will harvest a few drops of blood using the micro-capillary tube from one of the bleeding vessels prior to its clumping by the surgeon. After clumping of all the bleeding vessels, they are ligated using chromic catgut. The process of clumping and ligation of the bleeding blood vessels takes up to 3 minutes. The assistant surgeon will harvest blood samples from the bleeding vessel on the opposite end from where the surgeon starts to clump the first bleeding vessel. The actual process of sample collection will take less than one minute.

VMMC surgeons or their assistants will use capillary tubes to collect blood from the bleeding vessels and put directly on the blotting paper as described below. Details of the sample collection, preparation and laboratory procedures as well as SOPs are attached as Appendix 26 A & B which cover sample collection, preparation, laboratory procedures and SOPs for Unlinked HIV Testing.

Steps for DBS Sample from Bleeding Vessels:

1. Using heparinized capillary tubes, collect blood from the bleeding vessels immediately after circumcision
2. Fill the capillary tubes to almost ¾ with blood
3. Add 2 drops (50 µL) of blood in each circle of DBS sample collection card
4. Repeat procedure 3 until all the circles are completely filled.
5. Inspect card to ensure you have collected enough blood, and the specimen is valid
6. Place DBS sample collection card on a horizontal clean dry surface to **AIR DRY** for at least 3 hours
   - - 1. Procedure for DBS Sample from Finger Prick:
7. Let participant choose a finger to be pricked, preferably any of the three middle fingers.
8. Sterilize the chosen finger using alcohol swab and dry the finger using sterile dry swab.
9. Make a rapid and firm puncture of about 2-3mm deep to the palmar surface of the ring or middle finger (not at the side or tip of the finger) using a lancet, preferably BD Genie Lancet.
10. Wipe out the first drop of blood using sterile dry swab and collect subsequent free flowing blood or apply moderate pressure some distance above the puncture site to enable free flow of blood.
11. Proceed as in steps 2 to 6 of procedure *(i) “From Bleeding Vessels”* above
12. Stop the blood flow by applying slight pressure with a dry gauze pad or cotton wool at the punctured site.

These unlinked samples will be batched and taken to the laboratory, and results will be released in terms of number positive against number tested; the results will not be returned to the study sites. These measures will minimize the possibility of anyone linking the test result to the individual.

Blood samples from the field will be accompanied by laboratory request forms (Appendix 25: Sample Request/ Delivery Form) showing sample number, date of sample collection, Location and name of person collecting sample. The samples will be received by our study staff (WOA) seconded to the CDC/KEMRI HIV Research Laboratory in Kisumu. The staff holds a Higher National Diploma in Medical Laboratory Sciences and has undergone multiple short trainings in specialized Lab procedures. In addition, he has been trained on Good Clinical and Laboratory Practices and was the Laboratory Manager of a recently-closed Phase 3 randomized controlled clinical drug trial (FEM-PrEP, Bondo site, between 2009 and 2012) as well as the Kisumu Male Circumcision trial (2003-2009). We have made arrangement with the Kisumu CDC/KEMRI Laboratory Director [[5](#_ENREF_5)], also a co-investigator in this study, to train him ahead of the study start-up and monitor his performance in the course of the study.

Participants HIV Results:

Should they change their minds about knowing their HIV status, participants will have the option to obtain their HIV test results for up to one year following the date of the collection of the blood sample. Since the HIV testing will be done off-site (at the KEMRI/CDC laboratory), after consenting we will give each participant a slip of paper with his sample number and a telephone number that he can use to call and ask for his HIV test results. A Laboratory Technologist will be engaged by the study and trained to release results to any participant who calls to get his results. This person will be located off-site and will not be directly linked to the participants or the field staff who collect, label and ship the samples. In addition, given that the samples will not contain any identifiers, the Laboratory Technologist will not be able to link the test results to a participant. These measures will ensure that anonymity of participants is observed as initially planned. To verify that the person calling is the participant, the Laboratory Technologist will ask the caller for the month that the participant was circumcised and the name of the site where the circumcision took place. The sample number will be non-sequential random numbers. The sample numbers will not be linked or related to any numbers in the participant’s Participant Unique ID number used in the main study. Consequently if a participant loses this unique code, he will not be able to get his test results.

**Note:** The clinic staff obtaining specimens will not have access to the laboratory where the test will be done; similarly, the laboratory staff will not have access to the clinic records or meet the participants.

***Steps to follow when samples arrive at CDC/KEMRI Laboratory:***

1. Drivers or messengers shall deliver the samples from IRDO field stations to CDC HIV-R Laboratory weekly.
2. The samples shall be received by IRDO’s Lab staff, who will verify their state and accompanying request/delivery forms (e.g. labeling) and document in the specimen receiving book.
3. The samples shall be tested for HIV using CDC HIV-R Laboratory Specific SOPs as described under methods above and in the attached sample collection, preparation, laboratory procedures and SOPs (Appendices 26 A & 26 B).
4. The results shall be sent as a password-protected attachment from CDC HIV-R Laboratory to IRDO study Data Manager by email immediately they are ready (1 to 2 days for negative or positive results and one week for discordant results).

The results of this testing will affect future VMMC counseling for men who refuse testing. For example if the HIV rate among these men is significantly higher, then moving forward these males will receive increased counseling regarding the importance of testing, the lack of HIV prevention benefit if already HIV infected and they do not know, and the importance of condom use.

## Laboratory Procedure:

*Sample collection, Preparation, Laboratory procedures and SOPs for HIV testing* are attached as Appendix 26 A & B and include the following:

1. Preparation, Storage, Transportation and Processing of DBS for HIV Elisa Testing – Appendix 26 A
2. CDC HIV ELISA SOPs **–** Appendix 26 B

***Method validation***: We shall use DBS for HIV Elisa Testing under laboratory conditions, as described above and detailed in Appendix 26. KEMRI/CDC laboratory is ISO 15189 accredited and all the methods in the laboratory have been validated and operate as per the ISO standards (SOP for Method Validation is available in the KEMRI/CDC laboratory for reference).

***Biosafety training of study staff:*** All IRDO staff engaged in VMMC service provision are trained and experienced Clinical Officers (COs), Nursing Officers (NOs) and Infection Prevention Officers Thus, they are already trained on biosafety methods, with topics covering: precautions for collecting specimens; handling spills and accidents; handling and disposal of contaminated materials and waste; selection and care of gloves; sterilization and disinfection of medical equipment, etc. The COs and NOs will be trained on sample collection for HIV testing while biosafety refresher training for Lab staff will cover procedures for HIV testing and other topics above, as relevant.

**Disposal of testing materials:** The specimens obtained will be used only for Laboratory HIV testing in KEMRI/CDC laboratory, and once the results are out, the specimens will be immediately discarded and will not be used for any other purpose. Testing materials and other bio-wastes will be disposed of according to the MOH/WHO guidelines for VMMC service provision^37^. All our program staff are already trained on these guidelines and routinely dispose of such wastes. IRDO has a robust Quality Assurance/Quality Control system in place that provides routine monitoring of quality of VMMC and other clinical services. Our staff are also periodically supervised to ensure compliance with the WHO/MOH guidelines by internal and external experts (including those from the national and provincial MOH headquarters, as well as those from the local and international PEPFAR, CDC, USAID and DOD offices).

**COSTING VMMC IN THE CONTEXT OF THE INTERVENTIONS (SUB-STUDY 3)**

Study Design: One of the aims of the study is to determine the cost of demand creation, as well as the unit cost of providing VMMC services under each of the demand creation strategies being proposed. Data on costing will be collected across all the study arms. Whereas economic evaluation of service delivery has been conducted in several contexts ^[[32]](#endnote-31)^ there is limited information on cost-effectiveness of various approaches used for recruitment to increase service uptake. We plan to assess the cost-effectiveness of demand creation for VMMC services based on the three interventions. The results will demonstrate the accrued costs and net savings associated with each intervention strategy. This information will support policy and program decisions, guideline formulation, and allocation of scarce resources to scale up effective demand creation approach(es).

Methods:

The primary outcome of the main study is the incremental change in circumcisions performed attributable to the respective demand creation interventions. Costs will be estimated for primary outcome with reference to the control group, in which we will continue to provide routine recruitment approaches. This will demonstrate how the costs vary depending on the mode of demand creation.

We shall adopt a societal perspective because of the ability to integrate diverse factors into the model and the role the public sector plays in HIV prevention and management. This approach incorporates all costs borne by the implementers in recruiting participants for services.

## Sources of costing data

We will collect data from all study sites using a tool adapted from the Male Circumcision Decision-Makers’ Program Planning Tool (DMPPT) ^[[33]](#endnote-32)^ for each of the demand creation strategies (Appendix 22: Costing Instrument). The DMPPT was developed by the USAID/Health Policy Initiative in collaboration with UNAIDS to enable decision-makers to understand the potential cost and impact of various options for scaling up male circumcision services.^28^ The DMPPT has previously been used to undertake costing of male circumcision services in East and Central Africa.^[[34]](#endnote-33)^ We shall use this instrument as our data collection tool to estimate the cost of demand creation and the unit cost of providing VMMC (i.e. cost per adult circumcised) in each of the four study arms.

Table 6 below summarizes the key data sources for the various components of the unit costing tool and the DMPPT.

**Table 6:** Summary of data sources for use in costing

| **Type of Data** | **Source of Data** |
| --- | --- |
| Direct demand creation costs | - Requisitions from the stores to support demand creation. - Payments made by accounts department towards local transport for RAs, production and distribution of IEC materials, road shows, print and electronic media events, and other expenses. - The number of circumcisions done extracted from the VMMC registers. |
| Staffing and compensation | - Cost for hiring and training RAs will be based on actual training expenditure obtained from accounts and HR departments. - For staff involved in recruitment, their salaries, fringe benefits and allowances will be obtained from accounts department. |
| Operational costs | - The operational costs for each VMMC site will be derived from the records at the district level accounts offices. These include rent and utilities, vehicle insurance and maintenance, and communication costs. |
| Administrative and program costs | - We shall apportion the cost of supervision by key staff according to proportionate time spent on study related activities. Costs are salaries, allowances and fringe benefits. Other costs such as external audits, subscription fees to umbrella unions, and payments to statutory bodies shall be apportioned accordingly. |
| Service provision cost | - Service providers, field supervisors and support staff will be tracked in terms of salaries, allowances and fringe benefits; also, cost of VMMC supplies will be tracked, as well as proportional cost of equipment and instruments |

## Costing Data Analysis: Incremental cost-effectiveness ratio (ICER) estimation:

The cost effectiveness analysis will estimate the incremental costs (ΔC) and incremental effects (DALYs averted) (ΔE) attributable to IPC, DSO, IPC+DSO interventions, with reference to routine VMMC service delivery settings. Each will be expressed in terms of an incremental cost effectiveness ratio (ICER; ΔC/ΔE). ^29,^^[[35]](#endnote-34)^ The incremental cost-effectiveness ratios (ICERs) for each intervention shall be the key outcome measures of the model to determine the additional costs and benefits of each strategy over the other. We shall estimate the ICER for additional proportion of men aged 25-39 years accessing VMMC sites as the primary service delivery outcome. The ICER will be computed at a 3% discount rate (to account for time preference of cost and utility) using HIV infections averted and DALYs averted as the incremental benefits. ^[[36]](#endnote-35),^^[[37]](#endnote-36)^ This ratio demonstrates a range of plausible threshold (‘willingness–to–pay (WTP)) values of expenditure for the incremental benefits of respective intervention options.^[[38]](#endnote-37)^

The associated level of uncertainty will be characterized by estimating the cost-effectiveness acceptability curve (CEAC) for each intervention, using Monte Carlo simulations, which estimate the aggregate uncertainty from all inputs. Sensitivity analysis will be undertaken to assess the robustness of conclusions to key assumptions.^29^ We will use multivariate Monte Carlo regressions to estimate the differences in total cost between the settings and to adjust for potential confounders. We will attempt to identify what the cost drivers likely to be affected by the interventions are, how these items should be identified, and ascertain implications of uncertainty surrounding key drivers of cost.

We shall use a hypothetical cohort of 100,000 participants with similar characteristics to evaluate long-term outcome intervention effects using *Monte Carlo simulation approach*. The long-term outcome measures will include improved utility of/satisfaction level with services, improved uptake of services, and quality of outcomes. ^[[39]](#endnote-38),^^[[40]](#endnote-39)^

The incremental cost effectiveness ratio (ICER) of the three interventions will be based on the formulae provided below.

Where

The higher the ICER the less cost effective the intervention program is.

## Sensitivity Analysis

Variables included in the cost effectiveness analysis are not very stable because of parameter, methodological and structural uncertainties. Intuitively therefore, the ICER generated will have some degree of uncertainty, hence reducing its appeal for use in policy considerations. In view of this, sensitivity analysis will be conducted, to reveal the robustness of the ICER with respect to parameter changes. Scenario analysis involves changing the base case values with alternative values to examine changes in the ICER. The variables that can be changed include: overhead costs, staffing costs attributed to service delivery and variables for which there was the most uncertainty surrounding the resource use parameters.

# PHASE 5: ESTABLISHING ENDLINE MC UPTAKE/ESTIMATING IMPACT OF INTERVENTIONS

At Endline, we will review our Participants Master Log (Appendix 14) to identify those who did not go for VMMC at the 45 VMMC clinics participating in the study. We will use the telephone numbers obtained during enrollment to contact the participants. Those who say that they got circumcised but are not in our Participant Master Log, we will make an appointment and visit them at home, or another convenient location, to administer consent (Appendix 2C), conduct Endline interview (**Appendix 2D: Phone and In-Person Interview with Uncircumcised Men at Endline**) and verify their circumcision status. Those who report not having gone for VMMC will be asked why they did not go, and their response recorded in Appendix 2D. If more than one reason is given, the participant will be asked to rank the top three reasons from most to least important. As enrolled participants go for VMMC services, we will be recording in the study’s Participants Master Log that they have been circumcised. However, only those who go for VMMC at study-designated clinics where we have stationed Research Assistants to monitor uptake will be recorded in the Participants Master Log. Recognizing that some participants could choose to go for circumcision in non-study clinics, we will need to capture those ones as well. To do this, we will not call any person apart from men who are listed in our Participants Master Log. We will review VMMC program theater registers in non-study clinics located within the 12 districts where the study is being conducted and compare the names with those indicated on the Participants Master Log as having not gone for VMMC. For those where the three names of VMMC clients in theater registers match the three names in the Participants Master Log, we will contact them using the phone numbers registered in the Participants Master Log and NOT in VMMC theater registers. When calling, the TASCO participants who could have been circumcised at non-study clinics will be asked if they were enrolled in the study. If they were, we will schedule a visit at home and obtain consent and if willing, conduct endline survey and verify MC status; if not, they will not be contacted further.

Inclusion/Exclusion Criteria

Inclusion:

- - 1. Was enrolled in the main study
    2. Resident of the study village
    3. Did not go for circumcision per TASCO Study database

Exclusion:

1. Was not enrolled in the main study
2. Non-resident of study village

Has been circumcised per TASCO Study database

**Estimated study participant accrual period**

Following a review of recruitment data from numerous studies of VMMC (IRDO program data) that we have previously conducted in several sites in the study area some of which have been selected for study implementation in this application, we estimate a conservative average study-wide combined accrual rate of 822 eligible men (25-39 years) per month across the 45 sites (18-19 men per Location per month).

# DATA MANAGEMENT PLAN

IRDO has a well-developed data management office fully equipped with database systems for information management. The office supports all research and program activities for about 30 districts where IRDO is implementing VMMC in Nyanza and other parts of the country. These systems allow all sites to key information into the same main database and permit integration with a variety of different statistical applications such as STATA and SPSS. Data shall be entered directly into pass-word protected computers from paper copies by trained study data staff at the district level and linked to the central IRDO data management office (using the IRDO internal mail server). Paper copies will be secured in the data storage rooms with restricted access. Back up of electronic copies to external source shall be done daily with each update and secured by the data manager.

## Quality assurance of data entry

The data manager will periodically run data cleaning syntaxes to capture errors not detected by the in-built data quality controls. The systems also allow for rapid and accurate data collection and retrieval. Demographic, clinical and laboratory data will be collected by the study nurses or clinical officers using standardized questionnaires and entered directly into a centralized database from the sites. Before data entry, the questionnaires will be cross-checked by data supervisors for accuracy, and obvious errors corrected. Data forms quality will be assured in the following ways;

1. Paper forms will be reviewed for completeness before concluding visits with patients. The staff conducting interviews will check all the responses for missing or incorrect entries. The supervisors will also do random checks on forms for completeness and inconsistencies.
2. Paper forms with errors will be returned to the field staff to verify entries where possible.
3. Paper forms with errors will be presented during a bi-weekly study staff meeting to review errors and provide additional training to mitigate continued mistakes.
4. Data checks will be programmed to prevent entry of data values that are illogical, or when numeric values are entered rather than text and vice versa. The interviewer will be asked to check inconsistent and unlikely, but possible, responses (e.g., Age or marital status).

As part of quality control during data collection, we will engage research assistants to conduct data validation (go back to some sampled households to confirm the accuracy of the data collected previously). If, for example, a household previously documented as having no eligible men is found to have 2 eligible men during validation, the 2 eligible men will be listed and reached with the intervention; this will be done at the validation/listing visit or at a later day/time convenient to the participant. Similarly, if a household is found to have fewer eligible men during a data validation visit, then the enumeration data will be updated to reflect this change.

## Data storage and retention

Both hard and soft copy data will be stored in locked cabinets with limited access by data staff, technical support team, and CDC/KNH IRBs when indicated. To maintain privacy and confidentiality, participants will be identified using the Unique ID number. The code linking the ID number to individual identifying information will be kept in a separate secure location by the project coordinator. The ID number will be the only identifier captured on the data questionnaires and in the computerized database. Following completion of all data collection, the data forms will be archived at IRDO’s head office. One year following completion of the project, identifiers will be removed from the data. Hard copy tools will be retained for five years while electronic copies will be destroyed after ten years.

## Data Ownership and Sharing Policy

IRDO will own the project data, and the study PI, Co-PI and the funding agency (CDC) will have "rights" to access and use the data. The PI, Dr Kawango Agot, who is also the director of Impact-RDO, will have physical custody of the data on behalf of the organization. All users of IRDO data are required to acknowledge the sources of their data and to abide by the terms and conditions under which they accessed the original data.

## Data Analysis:

***Aim 1:*** ***Compare the rate of uptake of VMMC services by men aged 25-39 years exposed to Inter-Personal Communication, Designated Service Outlets, and IPC+DSO.***

The main outcome measure of this study is VMMC uptake. To assess the rate of uptake of VMMC services by uncircumcised men aged 25-39 years, we will compare the proportion of men who accept to be circumcised across the intervention arms. Specifically, the proportion of men accepting to be circumcised in each of the three arms will be compared to the proportion accepting circumcision in the no intervention group. P-values will be adjusted for multiple comparisons using the Bonferroni method. In order to take into account the clustered nature of the data, we shall also employ statistical methods suitable for analysis of cluster-randomized trials, namely the multi-level logistic regression model via the generalized estimating equations (GEE) approach with a random intercept. The random intercept term will represent the differences in true log-odds between the Locations. Odds ratio of VMMC uptake comparing intervention groups to the control group with the same underlying uptake rate will be estimated. This model will simultaneously assess individual-level and Location-level factors associated with uptake of VMMC as well as the effect of the interventions. Most importantly, it will enable the assessment of the intervention at the level at which randomization occurred: the Location level.

As outlined above under Phase 4, we shall conduct an endline survey in all households with men aged 25-39 years to establish the post-intervention MC uptake, and for those circumcised, collect information on when and where they were circumcised. These will be compared with similar data collected at baseline, using the methods described in the paragraph above. In addition, we shall extract from our database information on age of participants receiving services in each Location/Cluster. We will also have a sense of effectiveness of each recruitment strategy from surrendered referral coupons and from responses to question 1 in Appendix 20 B, asked to men who come for MC and through Appendix 2, the number of men reported and/or verified as circumcised at endline. This information, collected from multiple sources and through multiple approaches, will be compared across different arms of the study.

Program data will be routinely collected on the MoH VMMC client form (Appendix 23) and entered in IRDO data base at the facility level and submitted to the data coordinating office in Kisumu. This is the routine practice. The form has information on clients’ age, sub-location and district which we shall extract for men aged 25-39 years using the Data Extraction Form (Appendix 27). The extracted information will be used to document the ages of clients as well as the districts and sub-locations where they are coming from. We shall use the National 2009 Population Census Report to determine the corresponding Location and village for each sub-location.

***Aim 2: Determine the cost of providing VMMC among the three interventions relative to each other and to no intervention***.

To determine the cost of providing VMMC among the three interventions relative to each other and to no intervention, all expenses (equipment, medical supplies, office supplies, outreach costs, personnel, rent and utilities, local transport, etc.) related to the provision of VMMC will be tracked for every Location and calculated for each man. Cost-effectiveness of providing VMMC under the different interventions will then be assessed by comparing the average/median cost-per-man circumcised across the intervention arms. Detailed analysis plan is included under Costing VMMC in the context of the interventions above.

***Aim 3: Determine, through unlinked de-identified blood samples from bleeding vessels the proportion of men whose HIV status is unknown.***

HIV prevalence among men whose HIV status is unknown will be detected through testing of blood samples from the circumcision wound. HIV prevalence will be calculated by dividing the detected number of HIV by the total number of samples of men whose HIV status is unknown. The association between HIV sero-prevalence will be assessed using prevalence ratio (PR) and prevalence odds ratio (POR); 95% confidence intervals (CIs) will be computed for the ratios. Mantel-Haenszel and random effects model-based estimates of the adjusted prevalence ratios will be computed and logistic regression with random effects will be performed to obtain crude and adjusted prevalence odds of HIV for selected.

## Other Analyses:

*Other quantitative analyses*: Responses to questions on Appendix 20 B will be analyzed through simple descriptive statistics in terms of top barriers to and facilitators of VMMC uptake by age, intervention type, and study Location.

*Other Qualitative analyses:* Audio recordings will be transcribed and translated into English by study staff. The data analyst will then review to confirm accuracy and content validity based on the original audio-recordings. During the translation, we shall strive to keep the phrases and words as in the spoken language, to capture the context to the extent that is closer to the original language. The transcribed FGD and IDI narratives will be imported into the Atlas-ti software (version 5.0), for analysis. Transcripts will be read in their entirety before coding. Thematic content analysis of the data set will be performed with the discussion and interview guides being used to code each of the transcripts. Additionally in vivo codes will be identified during the coding. Participant experiences, views and recommendations shall be extracted from the text, condensed by abstraction of both overt and latent emphases as well as their unique experiences and expressions, and sorted out by relevant content areas. Conceptual categories related to communication or talking points on VMMC will be developed from the resulting themes.

## Data security

The following general security measures will be considered to ensure integrity of our data:

1. We will develop SOPs for data security and confidentiality procedures at collection, transfer, entry and storage levels, and make these readily accessible to all staff members who have access to confidential, individual-level data;
2. We will train staff on these procedures, including the use of passwords and codes to allow access to confidential information or data;
3. We will ensure all data are kept under lock and key, that access to confidential data is only limited to authorized persons, and that released data contains no identifiable information;
4. The study funder (PEPFAR and CDC) s well as the IRBs (KNH and CDC), will have unlimited access to data as long as they too comply with confidentiality conditions;
5. At the end of the study, data will be kept by IRDO for up to 10 years for electronic version and up to 5 years for paper forms, including consent forms. The PI (KA) will take responsibility of all study procedures, including long-term storage and disposition of data.

**Duration of Study:** The study will be in 45 Locations in 11 districts in former Nyanza province, and will take up to two years to complete.

**ETHICAL CONSIDERATIONS**

1. Ethical Review and Oversight: The proposal will be submitted to the Kenyatta National Hospital Ethics and Research Committee (KNH-ERC) and CDC Atlanta for ethical review and approval; KNH-ERC will also provide ongoing ethical oversight for the study; annual renewals will be obtained from both IRBs.
2. Potential risks and their Mitigation: Asking participants about their circumcision status and also verifying the same may be embarrassing to participants.

The RA will ask the participant to select a private location where MC status can be verified; we will do our best to keep study participants’ information private. However, there is a very small chance that someone outside of the research team may access information about the study – this is included in the consent document.

During sample collection for unlinked testing, we do not expect any new or unexpected adverse events outside of those normally occurring among persons receiving HTC and newly identified HIV-infected persons being enrolled in HIV care and treatment services.  These include negative reactions to an HIV diagnosis, incorrect test results, and breaches of confidentiality. There could also be a possibility of prolonged bleeding. If confidentiality is breached, this will be reported to the study coordinator within 24 hours of awareness and the coordinator who will report to the PI. The PI will send a written report to KNH-ERC via courier and to CDC IRB as email attachment within 24 hours of notification. Other protocol deviations (PDs) will be monitored by study staff and reported to the coordinator within 24 hours of receiving information;. All minor incidents that do not change the risk/benefit profile of participants (e.g. incorrect enumeration, listing of ineligible participants [not minors], etc.) – shall be reported with the annual IRB renewal. However, for more serious incidents and protocol violations that alter the risk/benefit profile of participants (e.g. enumerating in non-randomized villages, conducting interviews with ineligible participants), we shall continue to report any of those incidents immediately. The PIs will report urgent PDs within 48 hours and non-urgent ones annually, together with application for annual renewal. We have developed a tool (Appendix 28) for reporting incidents and protocol deviations.

The 2 questions in Appendix 20B will be asked, and IDIs (Appendices 9, 10, 12 & 13) conducted individually and in private. All respondents will be assured of confidentiality, that their names would not be written on the questions or reports, and that they are free to not answer any question they do not feel comfortable with and to withdraw from the study at any time. All those conducting enumeration of eligible participants and verifying their circumcision status will be male aged 25-39 years; they will be trained on how to approach the participants and how to ascertain the status. The procedure will take place in a private room identified by the respondent.

The blood sample for unlinked testing will be collected by the assistant surgeon while the surgeon is clamping and ligating the bleeding vessels hence the process of sample collection (which will take less than a minute) and will neither interfere with nor delay the process of achieving hemostasis or clumping of the blood vessels.

The sample label will only bear a pre-assigned serial number, date of sample and name of collector.

1. Compensation: Participants in the FGDs will be given actual fare refund of up to a maximum of 200/- and modest compensation for time taken to participate in the discussion, at KSh. 250/-. Since IDIs will take place where the participants are, no fare refund will be given, thus the total compensation for IDI participants will be KShs 250/-. Both at enrollment and endline surveys, participants will be given 250/- as compensation for time. The 250/- for compensation for time is below the minimum wage for a foreman in Kenya; which is Kshs. 324/- per day.
2. Benefits to Participants: All participants enrolled in the study will benefit by obtaining VMMC information contained in the booklet approved by the Ministry of Health (Appendix 17: *All you need to know about VMMC*). In addition, during recruitment, they will have a chance to ask questions and get answers from the RAs, especially those in the IPC and IPC+DSO Locations. They will be informed, during the consenting process that the information obtained from them will help the government of Kenya in getting older men to take up VMMC, an important factor in the fight against HIV acquisition in men and women.
3. Informed Consent: For those coming for VMMC and are 25-39 years, there will be written consent to ask them what influenced their decision to go for the services. Those in DSO and DSO+IPC intervention Locations who go for services in non-DSO sites will be asked why they still prefer these sites despite the availability of DSOs. Apart from the oral consent obtained only during household enumeration, for which we are requesting for waiver of documentation of consent, all consents for the study will be written. Before physical verification of VMMC status can be performed, written consent will be obtained by a male Research Assistant who is fluent in English and Dholuo.

For illiterate persons, an independent witness will be present for the reading of the entire consent and will also sign the consent form. A witness will be a person 18 years old who is literate, is a non-staff, and is accepted by the potential participant to witness the consenting process. Two copies of written consent forms will be signed by the person administering the consent and the participant (and where necessary, a witness); one copy will be stored under lock and key at the research office while another will be given to the participant to carry home for ongoing reference, and to get contact information of study and IRB leadership.

1. Confidentiality/Privacy: As is a requirement of all IRDO staff, all research staff will all undergo ethics training sign confidentiality agreement (Appendix 30: Confidentiality Statement). Research Assistants, VMMC service providers and data staff will be trained to ensure privacy during the data collection and consenting process. Data collection forms will be stored in the field and at IRDO head office in Kisumu, which will be the operating headquarters of the study. No participants will have any identifier on the data forms; names and signatures will only be on the consent forms, which will be kept under lock and key by the Study Coordinator and after signing, will be accessible only to the PIs, Coordinator and IRDO’s Research Officer (EO). Data will be entered in a computer that would be password protected and accessible only to the respective data clerk, the Data Manager, the PIs, the Coordinator and the Research Officer.

Participants will be given contact information of KNH-ERC and CDC IRB on the consent which they can refer to in case they want to report any violation of their rights or if they have any other study-related questions or concerns.

1. Study Staff Training in Handling Human Subjects

Regardless of prior training in research ethics, before protocol implementation, all study staff will undergo a three-day training on ethics and Good Clinical Practices (GCP), with topics covering: what is GCP, importance of GCP, goals of GCP, foundations for ethical conduct in research, the 13 principles of ICH-GCP, responsible persons for GCP compliance, and an overview in ethical issues in handling human subjects. This will be conducted by Kawango Agot (PI) in collaboration with officers at the Division of Global HIV/AIDS, CDC Kenya office. Subsequently all staff will be expected to undertake the online [Collaborative Institutional Training Initiative](https://www.citiprogram.org/default.asp) (CITI) pertaining to Human Subjects Research (basic or refresher course for those who have taken the course previously) as well as protocol-based training. The ethics training will be followed by protocol-based training.

1. Sponsor Monitoring: As the study sponsor, the Centers for Disease Control (CDC) may conduct monitoring/auditing of study activities to ensure the scientific integrity of the study and to ensure the rights and protection of study subjects. Monitoring and auditing activities may be conducted by:
   - CDC staff (“internal”)
   - Authorized representatives of CDC (e.g., a contracted party considered to be “external”).
   - Both internal and external parties.

Monitoring or auditing may be performed by means of on-site visits to the Investigator’s facilities or through other communications such as telephone calls or written correspondence. The visits will be scheduled at mutually agreeable times, and the frequency of visits will be at the discretion of CDC. During the visit, any study-related materials may be reviewed and the Investigator along with study staff should be available for discussion of findings.

The study may also be subject to inspection by regulatory authorities (national or foreign) as well as the IECs/IRBs to review compliance and regulatory requirements.

**EXPECTED APPLICATION OF RESULTS**

Results from the proposed study will inform programs currently implementing VMMC of the need to mount activities that would appeal to older men. The results will be shared with community leaders in study Locations and with various stakeholders in relevant forums, such as the national and provincial VMMC task-forces, and local and international meetings and conferences. In addition, findings will be shared through publications in peer-refereed journals.

**FLOW OF FORMS/APPENDICES**

**Table 7:** Summary Flow of Forms/Appendices

| **PHASE 1: HOUSEHOLD ENUMERATION** | | | |
| --- | --- | --- | --- |
| **Appendix** | | **Description** | **Purpose and flow** |
| Appendix 1: | | Household Enumeration Form – to capture information during household listing in all selected Locations/Clusters.  Obtain oral consent from the head of household or any adult member before initiating interviews | To capture: names of household heads; brief socio-demographic information; names of males age 25 – 39 years, their telephone numbers & time/day to revisit for MC ascertainment and interview; marital status; age, education level, occupation and religion of males >10 yrs.  Data manager to generate a list of eligible males and assign ID number to each – for use during revisits |
| **PHASE 2: CONDUCTING RAPID FORMATIVE STUDY** | | | |
| Appendix 4 | | Written Informed Consent for FGD with Circumcised and Uncircumcised) | Consenting circumcised and uncircumcised men for FGDs |
| Appendix 5 | | FGD Guide for Circumcised and Uncircumcised Men | To guide the conduct of FGDs |
| Appendix 6 | | Written ICF for IDI with Circumcised and Uncircumcised Men | Consenting circumcised and uncircumcised men for IDI |
| Appendix 9 & 12 | | IDI interview guide for circumcised men and partners of circumcised men | To conduct the IDIs with circumcised men and their female partners |
| Appendix 10 & 13 | | IDI Interview guide for uncircumcised men and partners of uncircumcised men | To conduct the IDIs with uncircumcised men and their female partners |
| **PHASE 3: ESTABLISHING BASELINE MC PREVALENCE AND PROPORTION OF ELIGIBLE MEN** | | | |
| Appendix 2A | | Written Informed Consent Form for Enrollment and Verification of Circumcision Status at baseline and endline | Use at post-enumeration revisit, RAs will: consent all men aged 25-39 years for enrollment; visually verification of MC status at baseline and endline. |
| Appendix 2B | | Enrollment Questionnaire and Circumcision Verification | Interview men aged 25-39 years on top reason(s) for going or not going for VMMC and verify MC status. |
| Appendix 3A | | Tracking Log. | A list of eligible men by location generated after household enumeration with information to be used for tracking them. |
| Appendix 29 | | Confidentiality Statement | For all study staff to indicate commitment to maintain confidentiality |
| **PHASE 4: IMPLEMENTING THE INTERVENTIONS** | | | |
| Appendix 14 | | Master Log | For collating enrollment information of all participants |
| Appendix 15 | | Referral Coupon | To refer eligible men for services. |
| Appendix 16 | | Toolkit for Addressing Barriers and Facilitators to VMMC | To be used for recruitment in IPC study arms |
| Appendix 18 | | DSO site information - places where s can find DSO sites in their respective Location | To guide s where dedicated VMMC services are offered |
| Appendix 17 | | MoH Approved VMMC Education Materials | To provide universal communication for recruitment for VMMC |
| Appendix 19 | | Monitoring Form for Demand Creation Strategies | To track communication strategies |
| Appendix 20A | | Written Consent Form for Two Questions With All VMMC Clients Aged 25-39 Yrs at Counseling | To consent all participants aged 25-29 years to be interviewed on motivators for VMMC. It will also be used to ask eligible males in DSO & DSO+IPC Locations seeking services in non-DSO sites why they opted for non-DSO sites.. |
| 20B | | Two Questions for All VMMC Clients Aged 25-39 Yrs at Counseling | To ask all participants aged 25-29 years 1 question to identify motivators for VMMC and 1 question to eligible males in DSO and DSO+IPC Locations seeking services in non-DSO sites To explore more about influencers of their choice for site |
| Appendix 21 | | Sample IRDO VMMC Flyer/Poster | Sample of flyer used for recruitment in all IRDO Locations which will be used in the control arms. |
| Appendix 22 | | Costing instrument | To collect information on cost of recruitment |
| Appendix 23 | | MoH VMMC Form | To extract information of eligible participants who are circumcised and identify s whose HIV status is unknown and potential for unlinked HIV testing. |
| Appendix 24: | | Written ICF for unlinked HIV testing on blood sample collected from bleeding vessels of participants whose HIV status is unknown | To obtain consent for unlinked HIV testing |
| Appendix 25: | | Sample request/delivery form | To record and track unlinked blood samples from the field to the KEMRI Lab in Kisumu |
| Appendix 26 A & B | | Procedures and SOPs for unlinked HIV testing | To be used for unlinked HIV testing of blood taken from participants with unknown HIV status. |
| Appendix 27: | | Data extraction form | To extract data from routine MoH and IRDO facilities |
| Appendix 28 | | Incident and Protocol Deviation Reporting Form | To be used for reporting any incidents or protocol deviations during the study |
| **PHASE 5: ENDLINE SURVEY** | | | |
| Appendix 2C | Written Informed Consent Form for Enrollment and Verification of Circumcision Status at endline | | To administer consent to collect end-line information during revisits to sampled households.  Endline information used to determine the number of men circumcised during the study. |
| Appendix 2B | Enrollment Questionnaire and Circumcision Verification | | Interview men aged 25-39 years on top reason(s) for going or not going for VMMC and verify MC status. |

**Table 8:** Proposed Timelines for Set Up and Data Collection

|  | **Description of activities** | **Months from Start** | | | | | | | | | | |
| --- | --- | --- | --- | --- | --- | --- | --- | --- | --- | --- | --- | --- |
|  |  | **1-3** | **4-6** | | **7-9** | | **10-12** | | **13-15** | **16-18** | **19-21** | **22-24** |
| **Start up** | Submit protocol to CDC Atlanta: CDC-DGHA Science Office; IRB. Select study locations, randomize to intervention arms. ***Up to 6 Months depending on final ethics approval*** | **x** | **x** | |  | |  | |  |  |  |  |
|  | Conduct desk review to develop messages addressing MC barriers and facilitators (use this to develop draft Demand Creation Information Tool); develop SOPs; identify local support structures, prepare logistics such as printing study materials. ***Up to 4 Months*** | **x** | **x** | |  | |  | |  |  |  |  |
| **Phase 1** | Hire and train staff, conduct household survey; visit all households with men aged 25-39 years; interview them; verify their MC status to establish MC prevalence and determine denominator. ***Up to 3 Months*** |  |  | | **x** | |  | |  |  |  |  |
| **Phase 2** | Recruit and train RAs  Conduct formative research (IDIs & FGDs).  ***1-2 Months*** |  |  | | **x** | |  | |  |  |  |  |
| **Phase 3** | Analyze information on barriers and facilitators of MC from formative phase; update and finalize Demand Creation Information Tool; submit tool to CDC and Kenyan IRBs for review and approval.  Conduct baseline interview and verification of MC status  ***Up to 3 Months unless ethics approval of modification delays***. |  |  | | **x** | | **x** | |  |  |  |  |
| **Phase 4** | Data analysis and integration of messages  Identify and train recruiters on study design and recruitment strategies.  ***Approximately 1 Month*** |  |  | |  | | **x** | |  |  |  |  |
|  | Implement the three interventions, including collecting information on costing and specimens for unlinked HIV testing. ***Approx. 9 Months*** |  |  | |  | | **x** | | **x** | **X** |  |  |
| **Phase 5** | Carry out endlline survey in all households with men 25-39 years uncircumcised at baseline; interview them; verify their MC status and use baseline data from men aged 25-39 years to determine the impact of the interventions. ***Approx. 3 Months*** |  |  | |  | |  | |  | **x** | **x** |  |
| **Cross-cutting** | Data entry, cleaning and analysis, report writing and manuscripts preparation. ***Ongoing up to 18 Months*** |  |  | | **x** | | **x** | | **x** | **x** | **x** | **x** |
|  |  | | | | | | | | | | | |
| **Sub-Study 4** | Develop SOPs; prepare logistics such as printing study materials. ***Approx. 2 Months*** |  | |  | |  | | **x** |  |  |  |  |
|  | Hire and train RAs. ***1 Month*** |  | |  | |  | | **x** |  |  |  |  |
|  | Enroll male participants in the study. ***Up to 6 Months*** |  | |  | |  | |  | **x** | **x** |  |  |
|  | Follow up male participants. ***Up to 8 Months*** |  | |  | |  | |  | **x** | **x** | **x** |  |
|  | Enroll female partners of the men in the study. ***Up to 6 Months*** |  | |  | |  | |  | **x** | **x** | **x** |  |
|  | Data entry and analysis; manuscripts preparation. ***Ongoing up to 12 Months*** |  | |  | |  | |  | **x** | **x** | **x** | **x** |

**KEY STAFF**

**Principal Investigator – Kawango Agot, PhD, MPH**; holds an MPH (Epidemiology) and a PhD (Medical Geography) degree from the University of Washington, Seattle. She is the Director of IRDO and Program Director of the two PEPFAR/CDC-funded HIV prevention, care and treatment programs in Nyanza: i) *Comprehensive and integrated HIV prevention interventions for a HIV-free Nyanza: The Pembetatu Model*, 3U2GPS002052 (2010-2015); and ii) *Tuungane II: Combination HIV prevention for Nyanza youth and general population*, 1U2GPS002777 (2010-2015). Dr. Kawango was also the Site PI of: i) *FEM-Prep Clinical Trial: A Phase 3, Multi-Center, Double-Blind, Randomized, Placebo-Controlled Effectiveness and Safety Study to Assess the Role of Truvada^®^ in Preventing HIV Acquisition in Women* (PI – L. van Damme, status); ii) *Site Preparedness and On-Going Community Activities for a Pre-Exposure Prophylaxis Clinical Trial* (PI – A. Corneli*);* and iii) *Sociobehavioral Research and Community Planning to Develop Site-specific Pilot Intervention Plans for PrEP Rollout* (PI – N. Mack). Between 2001 and 2007, Kawango was Study Coordinator and Co-Investigator of the Kisumu Circumcision Trial (PI – R.C. Bailey). Successfully implementing these and many other programs and research studies has accorded Dr. Kawango extensive experience with both research and program implementation, as well as necessary skills in maintaining ethical and professional conduct of high-visibility international programs and clinical trials. Dr. Kawango will provide overall oversight on all logistics of study.

**Co-Principal Investigator: Jonathan Grund, MA, MPH;** Mr. Grund is a Health Scientist in the Division of Global HIV/AIDS at the Centers for Disease Control and Prevention in Atlanta, USA. His work has focused extensively on HIV prevention topics including voluntary medical male circumcision, HIV testing and counseling, and vaccine policy research. He works closely with PEPFAR’s implementing partners providing male circumcision services on adverse event monitoring and reporting, external quality assurance, and program implementation. As a Co-PI, he will provide coordination and oversight for data analyses and conceptualization of publications, and act as the interface between IRDO and CDC Atlanta, as necessary. He will provide external technical support for quality assurance and review of data collection tools/methods as well as protocol and SOP development.

**Co-Investigator: Dr. Eunice Omanga, DrPH, MES**; is the head of research with IRDO. She received her Master’s degree in Environmental Health from University of Pennsylvania and DrPH from Drexel University, United States. She will provide direct oversight on the conduct of the study, supervise data entry, perform qualitative data analysis and support quantitative data analysis and manuscript writing.

**Co-Investigator, Naomi Bock, MD, MS;** is lead for the Biomedical Prevention team in the HIV Prevention Branch at the Division of Global HIV/AID at CDC. The team provides technical assistance for PEPFAR countries in areas of [1] biomedical prevention, including male circumcision, medical transmission (blood safety, injection safety, and waste management) and STI management; [2] programming to reach high risk, often hidden and marginalized groups engaged in high risk behaviors such as sex workers, men who have sex with men, and injection drug users; [3] multi-component programs to reduce alcohol-associated HIV sexual risk behaviors; and [4] developing, implementing, and evaluating programs to provide population coverage with an integrated package of HIV prevention services. Technical assistance areas include policy development and program planning, implementation and evaluation. She is author or co-author of more than 20 articles published in peer-reviewed journals. She received an MD from the University of Washington School of Medicine and a Masters of Science in Epidemiology from the Harvard School of Public Health. She will provide technical support during study implementation, review data for analyses, and participate in preparation of the publications.

**Study Coordinator: Jacob Onyango (BA, MA);** will be the Study Coordinator, overseeing day to day implementation of all aspects of the study. Jacob was the Study Coordinator of the Socio-Behavioral and Community Engagement component of the FEM-PrEP Clinical Trial. He also coordinated a number of Socio-Behavioral and HIV Prevention studied in the past.

**Co-Investigator and Study Statistician: Frankline Onchiri, BSc, MSPH, PhD(c);** holds a Bachelor’s degree in Mathematical Statistics from the University of Nairobi, Kenya, and an MSPH in Biostatistics and Epidemiology and PhD (c) in Biostatistics and Epidemiology, both from the University of Washington, Seattle. He will be charged with the overall statistical responsibilities for the main and sub-study and will be actively engaged in the design, conduct and final analysis of the data. Having participated in preparing the study design for this proposal, he will continue to give his input on study conduct up to the final data analysis, including manuscript preparation.

**Dr. June Odoyo, MBChB, MSc, PhD(c);** is the Technical Adviser, HIV Prevention at CDC. As the Activity Manager for VMMC for IRDO, Dr. Odoyo will provide technical oversight during the study

**Co-Investigator: Spala Ohaga, MSc, PhD*;*** is the Programs Manager at IRDO and deputizes the Director in overseeing the PEPFAR funded HIV prevention, care and treatment programs. He will work closely with the PI and other investigators as the link between the study and program activities to ensure that the study has access to requisite information including routine data.

**Co-Investigator: Samuel Mwalili (PhD);** is a Senior Statistician in Epidemiology and Surveillance with CDC, Kenya. He will work with FO to provide technical support with database creation, data management and analysis, and preparation of manuscripts for publication.

**Edward Mboya: BSc, MSc (Statistics);** is the Study Data Analyst. He will provide overall expert statistical support for the study and establish systems for routine analytic reports. He will also provide statistical support to the investigators in preparing presentations and manuscript for publication or conference presentation and the final study report.

**Co-Investigator:** **Julie Ambia, BScN, MPH, PhD (c);** is a doctoral candidate at the University of Nairobi. She will help with preparation of documents and reports needed before and during the study, including systematic review for demand creation messaging as well as manuscript write-up.

**Co-Investigator: Emily Zielinski-Gutierrez (MPH, DrPH);** is working with CDC-Kenya based in Kisumu. She will support the implementation of the protocol.

**Co-Investigator: Athanasius Ochieng’ (MBChB);** is the VMMC Manager at NASCOP. He holds a Bachelors degree in Medicine and Surgery from Moi University, Kenya, and has many years of experience in general surgery in various government health facilities in the country. In this study, he will provide technical support specifically with quality assurance, messaging for demand creation, and assure ongoing national support for the study. Will also participate in publications.

C**o-Investigator: Donath Emusu, MBChB, MPH, DrPH;** is a senior technical advisor, HIV Prevention, Division of Global HIV/AIDS at Centers of Disease Control and Prevention in Nairobi, Kenya. As an investigator, he will provide coordination oversight and liaison with CDC Atlanta and KEMRI for technical support. He will be available for consultation regarding day-to-day running of the study; he will also participate in site supervision and manuscript writing.

**Co-Investigator: Clement Zeh, PhD** Is the Director of KEMRI/CDC Kisumu Lab, and will provide oversight on lab procedures for HIV testing. He will also participate in manuscript preparation.

**Co-Investigator: Walter Otieno Agingu, HND (Med. Lab),** will perform HIV testing in the Lab, prepare SOPs for sample collection, storage and transportation (with CZ), train field staff, and participate in manuscript writing.

**Co-Investigator: Bernard Ayieko, HND, MCHD:** holds a Higher Diploma and Masters Degree both in Community Health and Development and a Diploma in Clinical Medicine & Surgery. He is the Project Coordinator of the VMMC Rollout under IRDO (CoAg# number: 3U2GPS002052). Ayieko was also a study clinician in the Kisumu VMMC RCT. He will work with Dr. Spala to oversee the interface between the study and program activities.

**Co-Investigator: Dr Boaz Otieno-Nyunya**: will provide both institutional and technical field support in implementing the protocol and also provide liaison with other CDC intervention activities in addition to supporting data processing and dissemination activities. He will also provide technical support for quality assurance and review of data collection tools/methods as well as protocol and manuscript development.

**Co-Investigator: Duncan Odera, MBChB, MPS (c);** is IRDO’s VMMC clinical Manager responsible for staff training and quality assurance and quality control of VMMC services. In the study, he will monitor quality and safety of services being provided to study participants going for VMMC.

**Co-Investigator –Leonard Soo, BSN, MPH, DrPH (c)**; will provide both institutional and technical field support in implementing the protocol and also provide liaison with other CDC intervention activities in addition to supporting data processing and dissemination activities. He will also provide technical support for quality assurance and review of data collection tools/methods as well as protocol and manuscript development.

**Co-Investigator: Lusi Ojwang, MBChB, MMED (Surgery),** is the Co-Chair to the Inter-County VMMC Taskforce (formerly, Nyanza Provincial VMMC Taskforce). He will provide technical oversight during surgery and collection of specimens for unlinked HIV testing.

**Consultants:**

**Dr. Urbanus Kioko, PhD (Health Economist**); is a senior lecturer in the department of Economics at the University of Nairobi; he will take charge of costing aspects of the study.

**Dr. Harsha Thirumurthy, PhD (Health Economist);** will work with Dr. Urbanus kioko to train staff, review cost analysis tools, data collection, analyze data and prepare manuscripts.

**Mr. Victor Ssempijja, MSc (Biostatistician);** **Investigator;** will provide technical support with data management and analysis, and preparation of manuscripts for publication.

**REFERENCES**

1. Auvert B, Taljaard D, Lagarde E, et al. Randomized, controlled intervention trial of male circumcision for reduction of HIV infection risk: the ANRS 1265 Trial. *PLoS Med* 2005; 2 (11):e298. Epub 2005 Oct 25. [↑](#endnote-ref-1)
2. Bailey RC, Moses S, Parker CB, et al. Male circumcision for HIV prevention in young men in Kisumu, Kenya: a randomised controlled trial. *Lancet* 2007; 369:643-56. [↑](#endnote-ref-2)
3. Gray RH, Kigozi G, Serwadda D, et al. Male circumcision for HIV prevention in men in Rakai, Uganda: a randomised trial. *Lancet* 2007; 369:657-66 [↑](#endnote-ref-3)
4. Weiss HA, Halperin D, Bailey, Robert C H, Richard J, Schmid G, Hankins C. Male Circumcision for HIV Prevention. From evidence to action? *AIDS* 2008, 22*:*567–574 [↑](#endnote-ref-4)
5. Siegfried N, Miller M, Deeks JJ. et al. 2009. **Male circumcision for prevention of heterosexual acquisition of HIV in men.** Cochrane Database of Systematic Reviews Reviews, 2009, Issue 2. Art. No.: CD003362 DOI: 10.1002/14651858.CD003362.pub2 [↑](#endnote-ref-5)
6. Male Circumcision Consortium 1. (MCC), M.C.C. 2011. News. December 2011; issue 33 [↑](#endnote-ref-6)
7. Hankins C, Hargrove J, Williams B, et al. Male circumcision for HIV prevention in high HIV prevalence settings: what can mathematical modelling contribute to informed decision making? *PLoS Med* 2009; 6: e1000109. [↑](#endnote-ref-7)
8. Weiss HA, Quigley MA, Hayes RJ. Male Circumcision and risk of HIV infection in sub-Saharan Africa: A systematic review and meta analysis. AIDS. 2000 Oct 20;14(15):2361-70. [↑](#endnote-ref-8)
9. www.malecircumcision.org [↑](#endnote-ref-9)
10. Amy Herman-Roloff, Robert Bailey, Kawango Agot, et al. (2010). A monitoring and Evaluation study to assess the implementation of male circumcision as an HIV prevention strategy in Kisumu and Nyando districts (MCMES). (Preliminary results) [↑](#endnote-ref-10)
11. Nelli Westercamp, Robert Bailey, Kawango Agot, at al. (2010). A Prospective Study of Behavioral Risk Compensation Related to Male Circumcision (MC) as an HIV Prevention Method (SHABS) (Ongoing). [↑](#endnote-ref-11)
12. National AIDS and STI control Programme. Kenya National strategy for Voluntary Medical Male Circumcision. Nairobi. Ministry of Public Health and Sanitation, Government of Kenya, 2009. [↑](#endnote-ref-12)
13. National AIDS Control Council 2. (NACC), N.A.C.C., *The Kenya National HIV/AIDS Strategic Plan 2010-2013 (KNASP III)*. 2009: Nairobi, Kenya.. (2009). The Kenya National HIV/AIDS Strategic Plan 2010 – 2013 **(**KNASP III). Ministry of Public Health and Sanitation, Nairobi, Kenya. [↑](#endnote-ref-13)
14. Nagelkerke, N., S. Moses, SJ de Villas, et al. (2007). "Modelling the public health impact of male circumcision for HIV prevention in high prevalence areas in Africa." *BMC Infect Dis* 7: 16. [↑](#endnote-ref-14)
15. Njeuhmeli E., S. Forsythe, J Reed et al. (2011). "Voluntary Medical Male Circumcision: Modeling the Impact and Cost of Expanding Male Circumcision for HIV Prevention in Eastern and Southern Africa." *PLoS Med* **8**(11): e1001132. [↑](#endnote-ref-15)
16. Bailey Robert C, Omar Egesah, Stephanie Rosenberg (2008). Male circumcision for HIV prevention: a prospective study of complications in clinical and traditional settings in Bungoma, Kenya. [*Bulletin of the World Health Organization*](http://www.who.int/entity/bulletin/en/). [Volume 86, Number 9, September 2008, 657-736](http://www.who.int/entity/bulletin/volumes/86/9/en/). [↑](#endnote-ref-16)
17. Nelli Westercamp, Robert Bailey, Kawango Agot, et al. (2010). A Prospective Study of Behavioral Risk Compensation Related to Male Circumcision (MC) as an HIV Prevention Method (SHABS) (Ongoing) [↑](#endnote-ref-17)
18. Nelli Westercamp, Mathew Westercamp, Thomas Reis, et al. Barriers and Facilitators of MC in adults: Evidence from recent studies in Nyanza Province. (Ongoing). [↑](#endnote-ref-18)
19. Matthew Westercamp, Robert Bailey, Kawango Agot, et al. Impact of Male Circumcision on Sexual Behaviors in Kisumu, Kenya. (Ongoing) [↑](#endnote-ref-19)
20. Renniel S, Muula A, Westreich D. Male circumcision and HIV prevention: ethical, medical and public health tradeoffs in low income countries. *J Med Ethics* 2007;33:357-361 doi:10.1136/jme.2006.019901 [↑](#endnote-ref-20)
21. Herman-Roloff A, Otieno N, Agot K, et al. (2011). Acceptability of Voluntary Medical Male Circumcision Among Uncircumcised Men in Kenya One Year After the Launch of the National Male Circumcision Program. PLoS ONE 6(5): e19814. doi:10.1371/journal.pone.0019814 [↑](#endnote-ref-21)
22. Ngalande RC, Levy J, Kapondo CP, et al. (2006) Acceptability of male circumcision for prevention of HIV infection in Malawi. AIDS Behavior 2006 10: 377–85. [↑](#endnote-ref-22)
23. Marya Plotkin,  Hawa Mziray, Jan Küver Judith Prince, et al (2011). Embe Halijamenywa: The unpeeled mango: A Qualitative Assessment of Views and Preferences concerning Voluntary Voluntary Medical Male Circumcision in Iringa Region, Tanzania. [↑](#endnote-ref-23)
24. Ivers, N. M., M. Taljaard, S. Dixon, et al. (2011). "Impact of CONSORT extension for cluster randomised trials on quality of reporting and study methodology: review of random sample of 300 trials, 2000-8." BMJ 343. [↑](#endnote-ref-24)
25. Study districts: K. East, K. West, Rongo, Homa Bay, Ndhiwa, Migori, Nyatike, Suba, Bondo, Rarieda and Siaya [↑](#footnote-ref-1)
26. KNBS (2010) 2009 Kenya Population and Housing Census, Volume I A; Population by Administrative Units, Kenya National Bureau of Statistics, Nairobi, Kenya [↑](#endnote-ref-25)
27. E.Odoyo-June , J H Rogers, W Jaoko, et al. Sex before wound healing and condom use among newly circumcised HIV-positive and HIV-negative men in Kisumu, Kenya. 19^th^ International AIDS Conference. Abstract no MOPE170 [↑](#endnote-ref-26)
28. Czarnogorski, M., Brown J, Lee V, et al. (2011). *The Prevalence of Undiagnosed HIV Infection in Those Who Decline HIV Screening in an Urban Emergency Department.* AIDS Res Treat, 2011. 2011: p. 879065. [↑](#endnote-ref-27)
29. Weinstock, H., Dale M, Linley L, et al., *Unrecognized HIV infection among patients attending sexually transmitted disease clinics.* Am J Public Health, 2002. 92(2): p. 280-3. [↑](#endnote-ref-28)
30. Dukers-Muijrers, N.H., et al., *Effectiveness of an opting-out strategy for HIV testing: evaluation of 4 years of standard HIV testing in a STI clinic.* Sex Transm Infect, 2009. 85(3): p. 226-30. [↑](#endnote-ref-29)
31. National AIDS and STI Control Programme NASCOP (2008). Adapted by the Male Circumcision Task Force, Ministry of Health, Kenya, from the WHO/UNAIDS/JHPIEGO *Manual for Male Circumcision under Local Anaesthesia.* Version 2.5C, February 2008. [↑](#endnote-ref-30)
32. Betrand JT, Njeuhmeli E, Forsythe S, et al. (2011) Voluntary Medical Male Circumcision: A Qualitative Study Exploring the Challenges of Costing Demand Creation in Eastern and Southern Africa. *PLoS ONE* 6(11):e27562.doi.10.137/journal.pone.0027562 [↑](#endnote-ref-31)
33. Bollinger L W, Plosky D, Stover J. (2009). Male circumcision: Decision Maker’s Program Planning Tool, calculating the costs and Impacts of a Male Circumcision Program. Washington, DC: Futures Group, Health Policy Initiative , Task Order 1 [↑](#endnote-ref-32)
34. Kahn JG, Marseille E and Auvert B. (2006) Cost-Effectiveness of Male Circumcision for HIV Prevention in a South African Setting. *PLoS Med* 3, e517. [↑](#endnote-ref-33)
35. Elizabeth, S., Stavros, P., Nishma, P., Jennifer, H., David, P., Maggie, R. and Peter, Blet al. (2012) Cost effectiveness of alternative planned places of birth in woman at low risk of complications: evidence from the Birthplace in England national prospective cohort study. *BMJ* 344. :e2292 [↑](#endnote-ref-34)
36. Gray, R.H., Li, X., Kigozi, G., Serwadda, D., Nalugoda, F., Watya, S., Reynolds, S.J. and Wawer, Met al. (2007) The impact of male circumcision on HIV incidence and cost per infection prevented: a stochastic simulation model from Rakai, Uganda. *AIDS* 21, 845-850 10.1097/QAD.0b013e3280187544. [↑](#endnote-ref-35)
37. Tekeste A, Wondafrash M, Azene G, et al. (2012) Cost effectiveness of community-based and in-patient therapeutic feeding programs to treat severe acute Malnutrition in Ethiopia. Cost Effectiveness and Resource Allocation 2012, 10:4 [↑](#endnote-ref-36)
38. Prinja, S., Bahuguna, P., Rudra, S., Gupta, I., Kaur, M., Mehendale, S.M., Chatterjee, S., Panda, S. and Kumar, R, et al. (2011) Cost effectiveness of targeted HIV prevention interventions for female sex workers in India. Sexually Transmitted Infections 87, 354-361. [↑](#endnote-ref-37)
39. David, B.E., Tessa Tan-Torres, E., Taghreed, A. et al. (2005) Methods to assess the costs and health effects of interventions for improving health in developing countries. *BMJ* 331, 1137-1140. [↑](#endnote-ref-38)
40. Loannidis, J.P.A. and Garber, A.M. (2011) Individualized Cost-Effectiveness Analysis. *PLoS Med* 8, e1001058. [↑](#endnote-ref-39)
